# Assessment of wall stresses and mechanical heart power in the left ventricle: Finite element modeling versus Laplace analysis

**DOI:** 10.1002/cnm.3147

**Published:** 2018-09-30

**Authors:** Matthias A. F. Gsell, Christoph M. Augustin, Anton J. Prassl, Elias Karabelas, Joao F. Fernandes, Marcus Kelm, Leonid Goubergrits, Titus Kuehne, Gernot Plank

**Affiliations:** ^1^ Institute of Biophysics Medical University of Graz Graz Austria; ^2^ Department of Mechanical Engineering University of California Berkley California; ^3^ Institute for Cardiovascular Computer‐assisted Medicine Charité ‐ Universitätsmedizin Berlin Berlin Germany; ^4^ Department of Congenital Heart Disease/Pediatric Cardiology German Heart Institute Berlin Berlin Germany

**Keywords:** aortic stenosis, heart failure, transvalvular pressure gradient

## Abstract

**Introduction:**

Stenotic aortic valve disease (AS) causes pressure overload of the left ventricle (LV) that may trigger adverse remodeling and precipitate progression towards heart failure (HF). As myocardial energetics can be impaired during AS, LV wall stresses and biomechanical power provide a complementary view of LV performance that may aide in better assessing the state of disease.

**Objectives:**

Using a high‐resolution electro‐mechanical (EM) in silico model of the LV as a reference, we evaluated clinically feasible Laplace‐based methods for assessing global LV wall stresses and biomechanical power.

**Methods:**

We used N = 4 in silico finite element (FE) EM models of LV and aorta of patients suffering from AS. All models were personalized with clinical data under pretreatment conditions. Left ventricle wall stresses and biomechanical power were computed accurately from FE kinematic data and compared with Laplace‐based estimation methods, which were applied to the same FE model data.

**Results and Conclusion:**

Laplace estimates of LV wall stress are able to provide a rough approximation of global mean stress in the circumferential‐longitudinal plane of the LV. However, according to FE results, spatial heterogeneity of stresses in the LV wall is significant, leading to major discrepancies between local stresses and global mean stress. Assessment of mechanical power with Laplace methods is feasible, but these are inferior in accuracy compared with FE models. The accurate assessment of stress and power density distribution in the LV wall is only feasible based on patient‐specific FE modeling.

**Abbreviations**
AVD: aortic valve disease
EM: electro‐mechanical
FE: finite element
HF: heart failure
IHP: internal mechanical heart power
IVC: isovolumetric contraction
IVR: isovolumetric relaxation
LV: left ventricle
PV: pressure‐volume.

## 1 INTRODUCTION

In stenotic aortic valve disease (AS), elevated pressure gradients impose a higher load upon the left ventricle (LV). Under such conditions, the pressure produced by the LV must increase in order to achieve an adequate cardiac output that meets the metabolic demands. This requires the LV wall to generate higher active forces, which can be achieved either by an increase in wall stresses or a change in ventricular shape and mass. Such pressure overload conditions, if persistent for long enough, trigger adverse remodeling processes, eventually precipitating progression towards heart failure (HF).^1^ Treatments aim at alleviating pressure overload by reducing transvalvular pressure gradients closer to normal levels by surgical‐ or catheter‐based aortic valve replacement.^2^ However, re‐stenosis frequently occurs, and despite a successful reduction of transvalvular pressure gradients, a majority of patients remains hypertensive, consequently showing increased risk for irreversible course of HF and higher morbidity and mortality.^3^ Thus, a successful reduction of pathologically elevated pressure gradients alone cannot be considered a reliable prognostic marker of long‐term post‐treatment outcomes in these patient cohorts. As a consequence, alternative biomarkers beyond pressure gradients are sought to that provide a complementary view of cardiac function and, potentially, offer a higher predictive power with regard to outcomes. In a recent study, the use of end‐diastolic or end‐systolic wall stresses as assessed by a wall stress index has been proposed as a novel diagnostic criterion of HF.^4^ This is physiologically motivated, as elevated wall stress levels are assumed to impair the balance between metabolic supply and demand^5^ by hindering perfusion and, thus, contribute towards adverse remodeling.^6^ Wall stresses are directly linked to the mechanical power generated by the myocardial muscle and the work performed by it and as such can be considered a metabolic marker. Different approaches have been proposed to assess work and the energy expenditure of the myocardium. As a direct measurement of energy metabolism, positron emission tomography (PET) was used(7, 8); however, the method is limited due to its complexity, including the need for tracers involving ionizing radiation. More recently, a concept of biomechanical internal myocardial heart power (IHP), necessary to maintain adequate cardiac output ( external heart power [EHP]), has been introduced in patients with aortic coarctation.^9^ Findings in this cohort suggest that the ratio EHP/IHP, referred to as power efficiency, improved mostly in those cases with elevated IHP. While potential marker qualities of such concepts need to be further evaluated, it has remained a yearned‐for goal to lower energy expenditure and increase efficiency of the myocardium likewise in any treatment procedure, including those for stenotic valvular and vascular disease.

Another method used to determine the work performed by the muscle is pressure‐volume (PV) relations. These are usually measured using conductance catheter techniques. However, these procedures are invasive, time‐consuming, and expensive. Alternatively, PV loops are measured with 3D echo or MRI,^10^ but even these methods are complex, and pressure and volume traces are not recorded simultaneously.

Despite the diagnostic potential of markers based on wall stresses and expended mechanical power, this assessment has not evolved towards a routinely used diagnostic tool in the clinic mainly due to methodological limitations. Attempts to address this relied upon different variants of Laplace law, which require the acquisition of only a small number of measures representing LV cavity volume, wall width, and pressure.(4, 9) However, these approaches are based on simplifications with regard to LV geometry, tissue structure, and biomechanical properties, as they assume the LV a thin‐walled mechanically isotropic spherical shell. The accuracy and validity of these simplifications have not been firmly established, thus casting doubt on the reliability and fidelity of any metrics based on them.(11, 12) Experimental validation based on direct measurements of stresses in vivo is challenging and not feasible yet with currently available technologies. However, an indirect inference is viable using computational tools such as FE modeling where 3D wall stresses can be computed from a set of reliable strains—either measured in vivo(13, 14) or computed in silico^15^ —using constitutive material models that are derived from ex vivo measurements^16^ and material parameters fitted to clinical data.(15, 17)

To evaluate the accuracy of Laplace analysis for estimating global wall stresses and mechanical power in the LV, we employed four FE‐based EM LV models that have been previously fitted and validated with clinical data^15^ under pretreatment conditions. These models provide reliable strain data at a high spatio‐temporal resolution from which wall stresses, biomechanical power, and work in the LV can be determined at the best possible accuracy. Laplace analysis was applied to these in silico models to estimate hoop stress and mechanical power over a cardiac cycle and compared with the global ground truth data based on FE analysis.

## 2 METHODS

### 2.1 Patient data

Data from four AS patients with clinical indication for aortic valve treatment, all preceding a cardiac magnetic resonance study, were used (Table 1). Stenotic aortic valve disease treatment indicators included valve area and/or systolic pressure drop across the valve. The study was approved by the institutional Research Ethics Committee following the ethical guidelines of the 1975 Declaration of Helsinki. Written informed consent was obtained from the participants' guardians.

**Table 1:** Pretreatment AS patient characteristics from MRI and noninvasive cuff pressure recordings including end‐diastolic volume (EDV), end‐systolic volume (ESV), stroke volume (SV), ejection fraction (EF), heart rate (HR), diastolic and systolic cuff pressures (p_dia_ and p_sys_), mean arterial pressure (MAP), wall thickness at the LV equator measured in septum/lateral free wall (h), pressure drop across aortic valve (Δp), presence of hypertension (HT), and mitral valve regurgitation (MVR)

	Sex	Age	EDV	ESV	SV	EF	HR	p_dia_	p_sys_	MAP	h	Δp	HT	MVR
		[years]	[ml]	[ml]	[ml]	[%]	[min^−1^]	[mmHg]	[mmHg]	[mmHg]	[mm]	[mmHg]	
A	F	63	112.0	46.0	66.00	58.93	53	74	126	91.33	12.0/12.5	95	No	No
B	M	73	121.0	54.7	66.32	54.81	81	75	134	94.67	11.2/13.8	62	No	Mild
C	M	54	118.2	42.2	76.14	64.42	75	71	141	94.33	16.0/18.2	79	Yes	Mild
D	M	85	172.0	103.0	69.00	40.12	68	79	144	100.67	14.0/15.2	59	Yes	No

### 2.2 Biomechanical FE model

The ventricular myocardium was modeled as a nonlinear, hyperelastic, nearly incompressible, and anisotropic material with a layered organization of myocytes and fibres that is characterized by a right‐handed orthonormal set of basis vectors.(16, 18) These basis vectors consist of the fiber axis **f**_0_(**x**), which coincides with the prevailing orientation of the myocytes at location **x**, the sheet axis **s**_0_(**x**), and the sheet‐normal axis **n**_0_(**x**). The mechanical deformation of the tissue is described by Cauchy equation of motion under stationary equilibrium assumptions leading to a quasi‐static boundary value problem: For a given pressure p(t), find the unknown displacement **u** such that

(1) $$\begin{array}{ll} -\Delta \cdot \sigma (u,t) & =0\;\;\;\;\;\;\text{in}\;\Omega \\ \sigma (u,t)\cdot n & =-p(t)\;n\;\text{on}\;\Gamma_{N}\\ \sigma (u,t)\cdot n & =0\;\;\;\;\;\;\text{on}\;\Gamma_{H}\\ u & =0\;\;\;\;\;\;\text{on}\;\Gamma_{D} \end{array}$$

holds for t ∈ [0,T]. By Ω, we denote the deformed geometry, and by Γ = ∂Ω, we define its boundary with $\Gamma =\bar{\Gamma_{D}}\;\cup \;\bar{\Gamma_{H}}\;\cup \;\bar{\Gamma_{N}}$ and $\left|\Gamma_{D}\right|>0$. The normal outward vector of Γ is denoted by **n**. The total Cauchy stress tensor **σ** refers to the sum of a passive stress tensor **σ**_pas_ and an active stress tensor **σ**_act_. That is, **σ**=**σ**_pas_ + **σ**_act_ with

(2) $$\sigma_{\text{pas}}=J^{-1}F\left(2\;\frac{\partial \Psi (C)}{\partial C}\right)F^{⊤},$$

(3) $$\sigma_{\text{act}}=J^{-1}F\left(S_{a}{\left(f_{0}\cdot Cf_{0}\right)}^{-1}f_{0}⊗f_{0}\right)F^{⊤},$$

where **F** is the deformation gradient, Ψ is the strain energy function, **f**_0_ is fiber orientation in the reference configuration, J =  det **F** is the Jacobian, **C** = **F** ^⊤^ **F** is the right Cauchy‐Green strain tensor, and S_a_ is the scalar active contractile stress generated by the myocytes acting along **f**_0_.

The passive behavior of myocardial tissue was modeled using two material models, either the transversely isotropic Guccione et al model,^16^

(4) $$\Psi_{\text{Gu}}(C)=\frac{\kappa }{2}{\left(\log\;J\right)}^{2}+\frac{a}{2}\left[\exp(Q)-1\right],$$

where

(5) $$Q=b_{f}{\left(f_{0}\cdot \bar{E}f_{0}\right)}^{2}+b_{t}\left[{\left(s_{0}\cdot \bar{E}s_{0}\right)}^{2}+{\left(n_{0}\cdot \bar{E}n_{0}\right)}^{2}+2{\left(s_{0}\cdot \bar{E}n_{0}\right)}^{2}\right]+2b_{\text{fs}}\left[{\left(f_{0}\cdot Ēs_{0}\right)}^{2}+{\left(f_{0}\cdot \bar{E}n_{0}\right)}^{2}\right]$$

and $\bar{E}=\frac{1}{2}(J^{-\frac{2}{3}}C-I)$ is the modified isochoric Green‐Lagrange strain tensor, or the isotropic Demiray model^19^

(6) $$\Psi_{\text{Dem}}(C)=\frac{\kappa }{2}{\left(\log\;J\right)}^{2}+\frac{a}{2\;b}\left\{\exp\left[b\;\left(\text{tr}(\bar{C})-3\right)\right]-1\right\},$$

with $\bar{C}=J^{-\frac{2}{3}}C$ the modified isochoric right Cauchy‐Green tensor. In both models, Equations 4 and 6, the bulk modulus κ, which serves as a penalty parameter to enforce near incompressibility, was chosen as κ = 650 kPa.

A simplified phenomenological contractile model^20^ was used to represent active stress generation:

(7) $$S_{a}=S_{\text{peak}}\;\phi (\lambda )\;\tanh^{2}\left(\frac{t_{s}}{\tau_{c}}\right)\;\tanh^{2}\left(\frac{t_{\text{dur}}-t_{s}}{\tau_{r}}\right)\;\text{for}\;0<t_{s}<t_{\text{dur}},$$

where S_peak_ is the peak isometric tension, φ(λ) is a nonlinear function dependent on fiber stretch $\lambda =\left|Ff_{0}\right|$ describing the length‐dependence of active stress generation, t_s_ is the onset of contraction, τ_c_ is the upstroke time constant, t_dur_ is the active stress transient duration, and τ_r_ is the downstroke time constant. This simplified model allows efficient fitting to patient data, as the parameters for peak stress, S_peak_, and time constant of contraction, τ_c_, and twitch duration, t_dur_, are related to the two clinical key parameters of interest, peak pressure, and maximum rate of pressure increase, in an intuitive manner.

Solving these equations under given mechanical boundary conditions using the FE method at a sufficiently high spatio‐temporal discretization provides an accurate description of tissue kinematics. Computed displacement **u** serve as input then in a postprocessing procedure to evaluate wall stresses **σ**(**x**,t) and to compute internal power expended by the LV (see section 2.6).

A Newton scheme was applied in each time step to linearize the nonlinear boundary value problem 1 yielding a nonsymmetric FE system. The linear FE system was solved by a parallel GMRES algorithm with an algebraic multigrid preconditioner. For the Newton scheme, a relative tolerance of 1.0e ^−5^ and an absolute tolerance of 1.0e ^−8^ were used as stopping criterion.

### 2.3 Verification of finite element model

To verify the FE‐based calculation of stress‐derived metrics, a geometrically simple and well‐studied benchmark problem was chosen for which circumferential hoop stresses can be found from Laplace law under the following assumptions:

(A1) The wall material is isotropic.
(A2) The shape is a symmetric spherical shell with inner radius, r, and outer radius, R.
(A3) The thickness of the wall, h = R − r, is sufficiently small, that is, the wall thickness to radius of curvature ratio is small, h/r ≪ 1.

**Figure 1:**
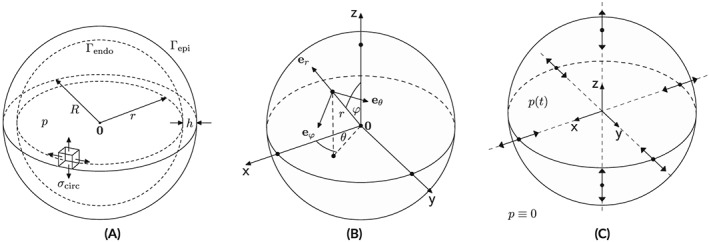
(A) Geometric setup, (B) sperical coordinate system and (C) displacement boundary conditions

Since all these assumptions are violated in the LV which is orthotropic **(A1)**, nonspherical in shape **(A2)**, and thick‐walled **(A3)** with h/r≈1, differences between Laplace analysis and FE computation are to be expected. Three configurations were considered, an ideal thin‐walled spherical shell, **Sph**_5_, which complies with all assumptions **(A1)**‐**(A3)** and thus can serve as a reference for FE validation, and two thicker‐walled spheres, **Sph**_25_ and **Sph**_150_, where assumption **(A3)** is increasingly violated. Geometries and mechanical boundary conditions are illustrated in Figure 1A‐C.

The inner radius was chosen as *r* = 15.0 mm in all models with *h* varying from *h* = 0.5 to *h* = 15.0 mm in **Sph**_5_ and **Sph**_150_, respectively. The choices for **Sph**_150_ are representative of the *h*/*r* ratios found in the LVs of patients in this study (Table 2). In line with assumption **(A1)**, the nonlinear isotropic material law stated in Equation 6 was employed with *a* = 10 kPa and *b* = 8. Passive inflation experiments were performed by solving 1 with ***σ***_act_≡0 and applying a pressure *p* in the range from 0 to 4 kPa to the endocardial surface, Γ_endo_, which covers the range of pressures observed in vivo during diastole. Pressure at the epicardial surface, Γ_epi_, was assumed to be zero. To render the solution of this pure Neumann problem unique, displacement boundary conditions were enforced at the intersections of the Cartesian axes with the epicardial surface by restricting displacements to the respective intersecting axes (see Figure 1C). Unstructured tetrahedral FE meshes were generated for the **Sph**_5_, **Sph**_25_, and **Sph**_150_ geometries where the mean spatial resolution, $\bar{d}x$, was increased until solutions were deemed converged.

**Table 2:** Geometric parameters inner radius, r, outer radius, R, wall thickness, h and wall thickness to radius of curvature ratio, h/r of spherical shell models **Sph**_5_, **Sph**_25_ and **Sph**_150_ and of image‐based anatomical LV models in the stress‐free reference configuration

	*r* [mm]	*R* [mm]	*h* [mm]	*h*/*r*
**Sph**_5_/**Sph**_25_/**Sph**_150_	15/15/15	15.5/17.5/30.0	0.5/2.5/15.0	0.033/0.166/1.0
**LV**_A_/ **LV**_B_/ **LV**_C_/ **LV**_D_	16.9/19.5/17.1/22.1	30.6/33.6/36.9/38.5	13.7/14.1/19.8/16.4	0.82/0.72/1.16/0.74

### 2.4 LV model

#### 2.4.1 Anatomical modeling

**Figure 2:**
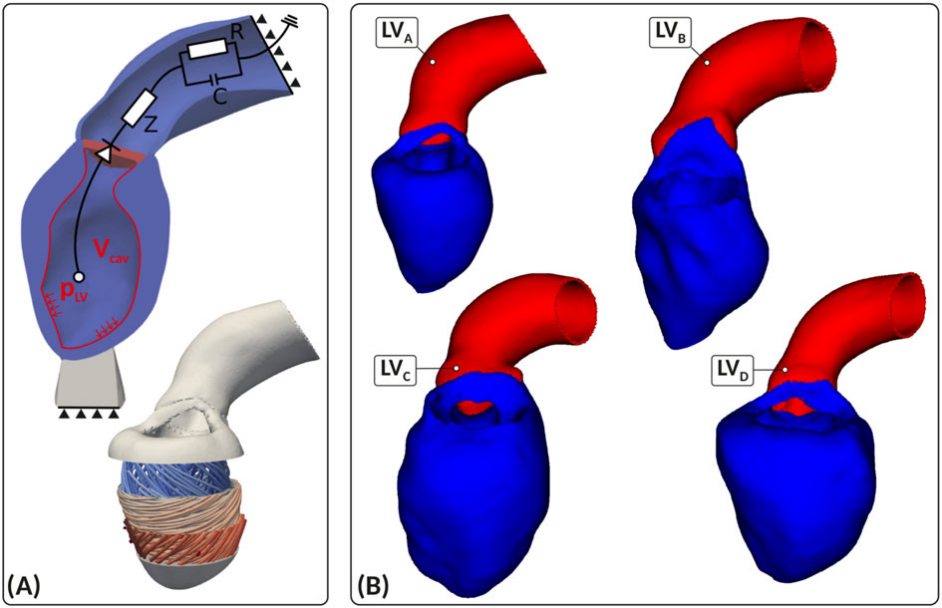
Image‐based patient‐specific left ventricle (LV) anatomy models. (A) Shown are the finite element model setup with Dirichlet (solid triangles) and Neumann boundary conditions controlled by a three‐element Windkessel model of afterload, and fiber architecture (bottom panel). (B) Patient‐specific anatomical models **LV**_A_, **LV**_B_, **LV**_C_, and **LV**_D_ of LV and aorta constructed from a 3DWH MRI scan in end‐diastolic configuration

Finite element meshes of the LV anatomy and aortic root were generated from 3D whole heart MRI acquired at end diastole (ED) with 1.458 × 1.548 × 2 mm resolution at the German Heart Center Berlin. Multilabel segmentation of the LV myocardium, LV cavity, and aortic lumen was done using the ZIB Amira software (https://amira.zib.de/ https://amira.zib.de/). Segmentations were smoothed and upsampled to a 0.1‐mm isotropic resolution.^21^ The wall of the aorta was automatically generated by dilation of the aortic lumen with a thickness of 1.2 mm, and the aorta was clipped before the branch of the brachiocephalic artery. Because of limited resolution, valves were not segmented but were included in the FE model as a thin layer of tissue for applying pressure boundary conditions and computation of cavity volume. The multilabel segmentations were meshed using CGAL (http://www.cgal.org/) with a target discretization of 1.25 mm in the LV myocardium and 1 mm in the aortic wall. For the transversely isotropic Guccione material model, see Equation 4, we equipped all models with a rule‐based fiber architecture,^22^ where fibers rotated linearly from −75° at the epicardium to +75° at the endocardium (Figure 2A). All anatomical models built are shown in Figure 2.

#### 2.4.2 Model fitting

To remove rigid body motion and provide physiological boundary conditions that allow a vertical movement of the LV base, as observed in vivo, mechanical boundary conditions were applied by fixing the terminal rim of the clipped aorta (Figure 2A) and resting the apex of the LV on an elastic cushion, which was rigidly anchored at its base. Constitutive relations were represented by Equation 4. Using the ED geometry, default material parameters and an estimated ED pressure (EDP), an initial guess of the stress‐free reference configuration was computed by unloading the model using a backward displacement method.(23, 24) Since clinically recorded data of the ED PV‐relation (EDPVR) are often limited, the Klotz relation^25^ providing an empiric description of EDPVR, *p*(*V*_cav_), was used as target to steer the fitting of constitutive parameters. In absence of accurate measurements of EDP, we refrained from fitting all material parameters to *p*(*V*_cav_). Rather, default values for the parameters *b*_*f*_ = 18.48, *b*_*t*_ = 3.58, and *b*_*fs*_ = 1.627 were used as reported in the literature,^16^ and only the scaling parameter *a* was adjusted individually for each patient. With a given data point (EDV, EDP) *a* was fitted to minimize the difference in stress‐free residual volume, *V*_0,dia_, between model and Klotz curve. This yielded values for *a* of 0.5, 0.65, 0.5, and 0.5 for the cases **LV**_A_, **LV**_B_, **LV**_C_, and **LV**_D_, respectively.

**Figure 3:**
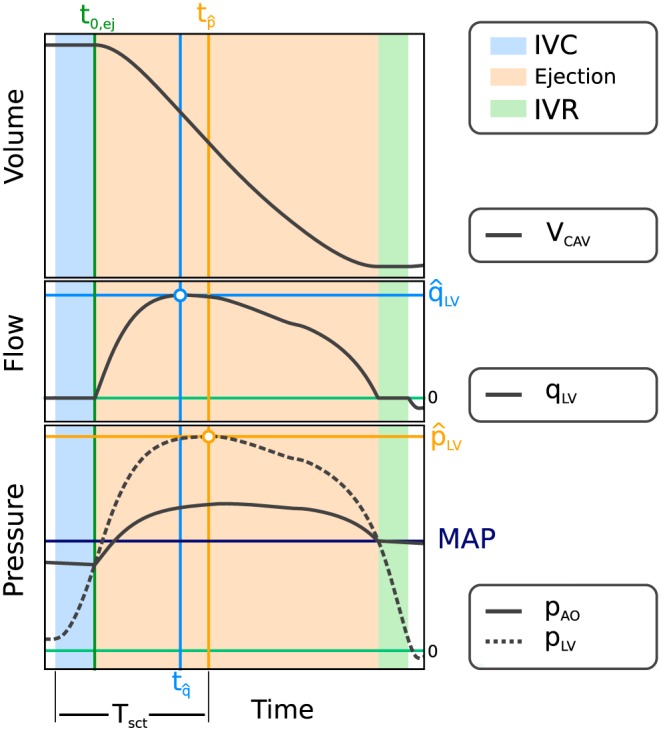
Fitting of the afterload model. Measured input data comprise V_cav_(t) (top panel), derived flow q_LV_ = d V_cav_/d t (mid panel), ${\hat{p}}_{\text{LV}}$ and pressure drop $\Delta p={\hat{p}}_{\text{LV}}-{\hat{p}}_{\text{ao}}$ along with simulated pressure traces p_LV_ and p_ao_ (bottom panel), with annotations of onset of ejection, t_0,ej_, instant of peak pressure in the left ventricle (LV), $t_{\hat{p}}$, cardiac contraction time, T_sct_, and the instant of peak flow, $t_{\hat{q}}$

A three‐element Windkessel model of LV afterload was used to provide the pressure‐flow relationship during ejection^26^ (see Figure 3). Left ventricle models were parameterized to match clinically recorded PV‐data using LV cavity volume traces, *V*_cav_(*t*), determined from Cine‐MRI with a temporal resolution of 45.28, 29.63, 32.00, and 35.29 ms for **LV**_A_, **LV**_B_, **LV**_C_, and **LV**_D_, respectively. Continuously monitored invasive pressure recordings were not available, as catheterization was not indicated. Peak pressure in the LV was determined by estimating peak pressure in the aortic root from cuff pressure measurements and by determining the pressure drop at peak flow across the aortic valve from ultrasound flow measurements using Bernoulli law.^27^ Windkessel parameters representing the aortic input impedance, *Z*, comprising the flow resistance of aortic valve, *Z*_*v*_, and the characteristic input impedance of the aorta, *Z*_c_, as well as resistance *R* and compliance *C* of the arterial system were fit to reproduce estimated LV peak pressure using measured volume traces *V*_cav_(*t*) as input.

In a final step, active mechanical properties were fit to the same hemodynamic data used for fitting the afterload model. A reaction‐eikonal model was used to generate activation sequences and simulate action potential propagation in the LV.^28^ Active stress generation was triggered with a prescribed electromechanical delay when the upstroke of the action potential crossed the −40 mV threshold. Parameters peak stress, *S*_peak_; time constant of contraction, *τ*_c_; and twitch duration, *t*_dur_, were adjusted manually to fit peak pressure, ${\hat{p}}_{\text{LV}}$, duration of pressure pulse and flow. Because of the intuitive link of the active stress model given in Equation 7 with the fitting targets, a satisfactory fit was achieved within ≤5 simulation runs. The goodness of fit was deemed sufficiently accurate when the clinically measured metrics EF, SV, MAP, and peak LV pressure, ${\hat{p}}_{\text{LV}}$, were matched within a margin of error of ±5*%*. Clinical input data and fitted model parameters are summarized in Table 3.

**Table 3:** Fitted parameters of circulatory, active stress, and passive mechanical model components

	Afterload				Active stress		Passive mechanical model
	$Z_{v}\left[\frac{kPa.ml}{ms}\right]$	$Z_{a}\left[\frac{kPa.ml}{ms}\right]$	$R\left[\frac{kPa.ml}{ms}\right]$	$C\left[\frac{ml}{kPa}\right]$	*S*_peak_ [kPa]	***τ***_*c*_ [ms]	*a* [kPa]
**LV**_A_	35.82	26.00	187.74	15.23	69	80	0.5
**LV**_B_	16.08	11.03	72.65	26.62	85	35	0.65
**LV**_C_	15.93	12.78	77.34	25.50	63	40	0.5
**LV**_D_	22.34	11.09	62.73	30.93	98	58	0.5

### 2.5 Myocardial wall stresses

Stresses can be computed from deformations **u** using constitutive material models based on ex vivo experimental data that link stresses to strains. In the FE model, stress tensors ***σ***(**x**,*t*) are computed by evaluating Equations 2 and 3, which yields a 3 × 3 tensor where only six components are independent for symmetry reasons. For the models **Sph**_5_, **Sph**_25_, and **Sph**_150_, the stress tensor simplifies. Because of the assumption of isotropy in **(A1)** and symmetry in **(A2)**, any solution, if expressed in a spherical coordinate system, must also be symmetric. Quantities computed in the FE Cartesian coordinate system are recast in spherical coordinates as defined in Figure 1B using a projection matrix, **P**. For the total Cauchy stress tensor ***σ***, we obtain

(8) $$\sigma =\left(\begin{array}{ccc} \sigma_{xx} & \sigma_{xy} & \sigma_{xz}\\ \sigma_{yx} & \sigma_{yy} & \sigma_{yz}\\ \sigma_{zx} & \sigma_{zy} & \sigma_{zz} \end{array}\right)=P\left(\begin{array}{ccc} \sigma_{rr} & \sigma_{r\phi } & \sigma_{r\theta }\\ \sigma_{\phi r} & \sigma_{\phi \phi } & \sigma_{\phi \theta }\\ \sigma_{\theta r} & \sigma_{\theta \phi } & \sigma_{\theta \theta } \end{array}\right)P^{⊤}=P\;\sigma_{\text{sph}}\;P^{⊤},$$

with the projection matrix **P** = (**e**_*r*_,**e**_*ϕ*_,**e**_*θ*_) and $r\in R^{+},\theta \in [0,\pi ],\phi \in [0,2\;\pi )$.

While all quantities in the spherical models must be perfectly symmetric, this is not necessarily the case in the FE solutions. Depending on spatial resolution and boundary conditions, a minor numerical jitter around mean values will inevitably occur. For comparing FE with Laplace analysis, averaged mean quantities were therefore computed over the entire domain by

(9) $${\bar{\sigma }}_{⋆⋆}(u,t)=\frac{1}{\left|\Omega \right|}\;\int_{\Omega }\sigma_{⋆⋆}(u,t)\;dx,$$

with **u** being the FE solution at time *t* and ⋆ ∈ {*r*,*θ*,*ϕ*}.

In a thin‐walled spherical shell, stresses in azimuthal and meridional direction must be equal due to symmetry, that is, *σ*_circ_ = *σ*_*ϕϕ*_ = *σ*_*θθ*_, and with *h* ≪ *r*, radial stresses can be assumed to be negligible relative to circumferential hoop stresses, that is, 0≈*σ*_*rr*_ ≪ *σ*_circ_. The stress tensor in spherical coordinates simplifies therefore to

(10) $$\sigma_{\text{sph}}\approx \left(\begin{array}{ccc} 0 & 0 & 0\\ 0 & \sigma_{\text{circ}} & 0\\ 0 & 0 & \sigma_{\text{circ}} \end{array}\right).$$

As a reference for verifying FE‐based stresses, different variants of Laplace law were used. In particular, we use an extension of Laplace law that takes into account the finite thickness of the wall

(11) $$\sigma_{\text{circ}}=\frac{p\;r}{2\;h\left(1+\frac{h}{2\;r}\right)}=\sigma_{\text{L,H}}$$

and refer to stress estimates based on this formula as Laplace stress in thick‐walled spherical shells, *σ*_L,H_. Exploiting assumption **(A3)**, that is, $\frac{h}{r}\ll 1$ and thus $\left(1+\frac{h}{2\;r}\right)\approx 1$, allows a further simplification yielding

(12) $$\sigma_{\text{circ}}=\frac{p\;r}{2\;h}=\sigma_{\text{L,h}},$$

which we refer to as Laplace stress in thin‐walled spherical shells, *σ*_L,h_. Finally, we consider a volume‐based estimation of *σ*_circ_ 29 defined as

(13) $$\sigma_{\text{circ}}=\frac{p}{{\left(\frac{V_{\text{cav}}+V_{\text{myo}}}{V_{\text{cav}}}\right)}^{2/3}-1}=\sigma_{\text{L,V}},$$

which has been used previously in clinical studies.^4^ We note that Equation 13 is equivalent to Equation 11 for a spherical shell. However, when applied to a nonspherical structure such as the LV, this is not the case. Equation 13 may offer advantages, as the determination of *V*_cav_ and *V*_myo_ may be less ambiguous than the determination of a representative inner radius *r* and wall thickness *h* (see section 2.8.1).

### 2.6 Myocardial power and work

For a given displacement **u** at time *t* ∈ [0,*T*], where *T* refers to the duration of a cardiac cycle and *t*_0_ = *t*_ED_ = 0 marks the end of diastole, the biomechanical power density, *p*_int_, generated or consumed at location **x** within the LV wall can be computed by evaluating

(14) $$p_{\text{int}}(x,t)=\sigma (u,t):\dot{\varepsilon }(u,t),$$

where $\dot{\varepsilon }$ is the strain rate tensor and **A**:**B** = tr(**A** ^⊤^ **B**) denotes the double contraction of two tensors; see, eg, Holzapfel^30^ for further details. Integration of Equation 14 over the entire myocardial wall yields the global biomechanical power, *P*_int_,

(15) $$P_{\text{int}}(t)=\int_{\Omega }\sigma (u,t):\dot{\varepsilon }(u,t)\;dx,$$

and integration of Equation 15 over time yields an expression of biomechanical work, *W*_int_, performed

(16) $$W_{\text{int}}=\int_{t_{0}}^{t}P_{\text{int}}(\tau )d\tau .$$

Based on Laplace law, biomechanical power can be estimated using

(17) $$P_{\text{int},⋆}(t)=V_{\text{myo}}(t)\;\sigma_{L,⋆}(t)\;\underset{\approx {\dot{\varepsilon }}_{\text{circ}}}{\underbrace{\left(\frac{\dot{r}(t)}{r_{0}}+\frac{\dot{R}(t)}{R_{0}}\right)}},$$

where ⋆ denotes which formula was used for estimating the circumferential wall stress that is, $⋆\in \left\{h,H,V\right\}$, and ${\dot{\varepsilon }}_{\text{circ}}$ approximates circumferential strains. For a derivation of Equation 17, see Supporting Information. Laplace‐based mechanical work is estimated analogously to Equation 16, yielding

(18) $$W_{\text{int},⋆}(t)=\int_{t_{0}}^{t}P_{\text{int},⋆}(\tau )\;d\tau .$$

In addition, a recently introduced also Laplace‐based relative power indicator, IHP, was evaluated, which attempts to estimate the power generated by the LV around the instant of peak pressure, $t_{\hat{p}}$. Based on Preston and Wilson,^31^ the mechanical work expended, or internal mechanical heart work (IHW), during contraction time, *T*_sct_, defined as the time elapsed between the onset of isovolumetric contraction (IVC) at *t*_ED_ and the instant of peak stress in the LV at $t_{\hat{p}}$, is approximated by

(19) $$\text{IHW}=V_{\text{myo}}\;\sigma_{L,⋆}.$$

IHW is interpreted as a measure of the mechanical potential energy stored in the LV from which a measure of the peak biomechanical power generated by the LV between *t*_ED_ and $t_{\hat{p}}$ is derived then by

(20) $$\text{IHP}=\frac{\text{IHW}}{T_{\text{sct}}}.$$

Note that Equation 20, in contrast to Equation 17, does not include any measure of $\dot{\varepsilon }$. Thus, while consistent in terms of physical units, IHP must be considered a relative indicator and not a physical measure of power.

### 2.7 Hydrodynamic power and work

Hydrodynamic power, *P*_ext_, is given by

(21) $$P_{\text{ext}}=p\;q=p\;\frac{dV_{\text{cav}}}{dt},$$

where *p* is the hydrostatic pressure acting at endocardial surface, Γ_endo_, and *q* represent blood flow out of the LV cavity during ejection. Hydrodynamic work, *W*_ext_, is then the work expended by the LV myocardium when changing the volume of its cavity, *V*_cav_, given by

(22) $$W_{\text{ext}}=\int_{t_{0}}^{t}p(\tau )\;q(\tau )\;d\tau ,$$

or, equivalently, expressed as PV work by

(23) $$W_{\text{ext}}=\int_{V_{{\text{cav}}_{0}}}^{V_{{\text{cav}}_{1}}}p(V_{\text{cav}})\;dV_{\text{cav}}.$$

In absence of active stresses, ie, ***σ***_act_ = 0, and isovolumetric contstraints imposed by valves upon *V*_cav_, *P*_int_≡*P*_ext_ must hold. Under such conditions, external work can therefore serve as a reference for validating the FE‐based computation of internal power and work. This is not necessarily the case during the isovolumetric phases of a heartbeat where internal work may be expended, which does not necessarily manifest as external work. During these phases, changes in ***σ*** occur, which may entail shape changes of the LV myocardium and thus induce a nonzero strain rate tensor $\dot{\varepsilon }$. However, because of the isovlumetric constraints imposed by the incompressibility of the blood pool and the closed state of all valves, no global change in cavity volume can occur, ie, d*V*_cav_ = 0.

Under healthy conditions, hydrodynamic power in the LV cavity equals the power delivered to the arterial system, as transvalvular power losses are small. However, in AS cases where transvalvular pressure gradients, Δ*p*, are significant, the effective hydrodynamic power externally delivered to the arterial system is reduced. Following Fernandes et al,^9^ we define external hydrodynamic heart power, EHP, as

(24) $$\text{EHP}={\bar{P}}_{\text{ext,ao}}=\frac{1}{T_{\text{sys}}}\int_{t_{\text{ED}}}^{t_{\text{ES}}}p_{\text{ao}}\;q\;dt\approx \text{MAP}\cdot \text{CO},$$

where MAP and CO are mean arterial pressure and cardiac output, respectively, and *p*_ao_ = *p*_LV_ − Δ*p* is the pressure in the aorta ascendens. Power efficiency, *P*_eff_, has been defined previously in Fernandes et al^9^ as the ratio

(25) $$P_{\text{eff,clin}}=\frac{\text{EHP}}{\text{IHP}}.$$

Since *P*_eff_ essentially relates the mean hydrodynamic power delivered to the arterial system to the peak biomechanical power generated by the LV myocardium during systole, *P*_eff_ can be expressed as

(26) $$P_{\text{eff}}=\frac{{\bar{P}}_{\text{ext,ao}}}{{\hat{P}}_{\text{int}}}\approx \frac{{\bar{P}}_{\text{ext,ao}}}{{\hat{P}}_{\text{ext}}}.$$

where ${\hat{P}}_{\text{int}}$, ${\hat{P}}_{\text{ext}}$, and ${\bar{P}}_{\text{ext,ao}}$ are determined based on Equations 15, 21, and 24, respectively. That is, *P*_eff_ can be estimated from hemodynamic data using ${\hat{P}}_{\text{ext}}$ or from LV deformation analysis using ${\hat{P}}_{\text{int}}$. Unlike *P*_eff,clin_ in Equation 25, which compares an absolute measure of external hydrodynamic power to a relative indicator of internal biomechanical peak power, Equation 26 provides a physically consistent comparison.

### 2.8 Evaluation of Laplace‐based assessment of wall stress and power

Human EM LV models that were validated against clinical data (see section 2.4.2), provided accurate ground truth data on strains ***ϵ***(**u**,*t*) and stresses ***σ***(**u**,*t*) in the LV wall. Using these as reference, Laplace analysis was applied to the in silico models to assess its accuracy and validity.

#### 2.8.1 Determination of clinical input data for Laplace‐based analysis

Geometric input parameters *r* and *h* required for Laplace analysis must be determined from clinical imaging datasets. As LV shape deviates markedly from a spherical shell, representative mean parameters of *r* and *h* must be found. Since there is no unique best solution to establish a geometric correspondence between LV shape and a spherical shell, various methods have been used in clinical applications. Typically, transverse slices from short‐axis Cine‐MRI scans were analyzed to measure, either manually or semiautomatically, *r* and *h*, where *h* is measured in the postero‐lateral wall, the septum or an average is taken. The analysis is either carried out in one representative mid‐cavity LV short axis slice, or a number of slices is selected to capture representative basal, mid‐cavity, and apical LV cross sections.

**Figure 4:**
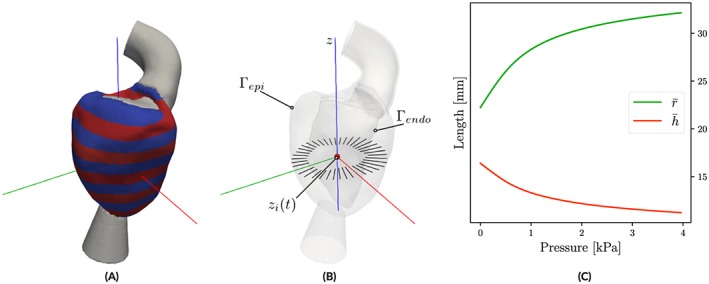
Determination of input parameters radius $\bar{r}$ and wall width $\bar{h}$ for Laplace analysis. (A) Automated left ventricle slicing along long‐axis **z**. (B) Sampling of variations in r, R, and h within a slice, z_i_. (C) Averaged parameters $\bar{r}$ and $\bar{h}$ as a function of p

Similar issues arise when applying Laplace analysis to in silico datasets. In order to extract *r* and *h* as objectively as possible without operator bias, automated processing workflows were implemented (see Figure 4). Analogous to the *z*‐slice selection in MRI protocols, the unstructured FE meshes of the LV models were decomposed into slices of ≈8 mm resolution, comparable with the MRI out‐of‐plane resolution. Decomposition was achieved by first determining the long axis, **z**, of the LV using principal component analysis (see Figure 4A), which yielded, depending on the spatial extent of the LV long‐axis of a given model, between 10 and 14 slices; each slice *i* is centered around *z*_*i*_. A mean *z* coordinate of the LV in its current configuration, ${\bar{z}}_{c}(t)$, was computed to define the center slice plane using the long‐axis unit vector, *e*_z_, and the center *z*_*i*_(*t*) of individual slices was shifted, keeping slice width and distance to the LV center ${\bar{z}}_{c}(t)$ constant. Within a selected plane, radial vectors, **r**_*i*,*j*_(*t*), were computed that emanated from *z*_*i*_ and were oriented in polar angles *ϕ*_*j*_ ranging from 0° to 360° with an angular sampling of Δ*ϕ* = 9°. For each vector **r**_*i*,*j*_(*t*), the intersection with surfaces, Γ_endo_(*t*) and Γ_epi_(*t*), was determined, yielding *N* = 2*π*/Δ*ϕ* inner radii, *r*_*i*,*j*_(*t*); outer radii, *R*_*i*,*j*_(*t*); and wall widths, *h*_*i*,*j*_(*t*) = *R*_*i*,*j*_(*t*) − *r*_*i*,*j*_(*t*) (Figure 4B). Mean radius, ${\bar{r}}_{i}(t)$, and wall width, ${\bar{h}}_{i}(t)$, were determined as the arithmetic average

(27) $${\bar{r}}_{i}(t)=\frac{1}{N}\sum_{j=1}^{N}r_{i,j}(t)\;\text{and}\;{\bar{h}}_{i}(t)=\frac{1}{N}\sum_{j=1}^{N}h_{i,j}(t).$$

Finally, multislice mean $\bar{r}$ and $\bar{h}$ were computed by averaging over *M* slices

(28) $$\bar{r}(t)=\frac{1}{M}\sum_{i=1}^{M}{\bar{r}}_{i}(t)\;\text{and}\;\bar{h}(t)=\frac{1}{M}\sum_{i=1}^{M}{\bar{h}}_{i}(t).$$

The time course of the mean values $\bar{r}(t)$ and $\bar{h}(t)$ (see Figure 4C) was plugged then into the respective Laplace equations to compute stress in Equations 11, 12, and 13; power in Equation 17; and work in Equation 18.

#### 2.8.2 Simulation protocols and data analysis

To evaluate the influence of violating the assumptions **(A1)** and **(A2)**, passive inflation experiments were performed with LV models and the anisotropic material given in Equation 4 following the same protocol as applied before to the spherical shell models **Sph**_5_, **Sph**_25_, and **Sph**_150_ in section 2.3. Laplace‐based stress estimates *σ*_L,h_, *σ*_L,H_, and *σ*_L,V_ were compared with the mean stresses obtained from the FE solution. Stresses were evaluated with respect to an ellipsoidal coordinate system to facilitate a comparison with stresses in the spherical shell models (see Figure 1B). An ellipsoidal coordinate system was constructed for the LV models by assigning fiber and sheet orientations using a rule‐based method with a constant fiber angle of 0°.^22^ Stress components *σ*_*rr*_(**x**), *σ*_*ϕϕ*_(**x**), and *σ*_*θθ*_(**x**) were averaged according to Equation 9, yielding ${\bar{\sigma }}_{rr}$, ${\bar{\sigma }}_{\phi \phi }$, and ${\bar{\sigma }}_{\theta \theta }$, respectively. Laplace‐based estimation of power, *P*_int,⋆_, was compared with FE‐based power, *P*_int_, and to external hydrodynamic power in the LV cavity, *P*_ext_.

Laplace analysis was applied to clinically fitted EM LV models **LV**_A_–**LV**_D_ to compare LV stress *σ*_L,⋆_; power *P*_int,⋆_; and IHP over an entire systolic cycle to the FE‐based stresses ${\bar{\sigma }}_{rr}$, ${\bar{\sigma }}_{\phi \phi }$, ${\bar{\sigma }}_{\theta \theta }$, and ${\bar{\sigma }}_{\text{mean}}$ and biomechanical power *P*_int_. Further, biomechanical power due to LV deformation, *P*_int_, and hydrodynamic power, *P*_ext_, derived from PV data were also compared to assess differences during isovolumetric phases.

### 2.9 Numerical solution

Discretization of all PDEs and the solution of the arising systems of equations relied upon the Cardiac Arrhythmia Research Package framework.^32^ Details on FE discretization^33^ as well as numerical solution of electrophysiology(28, 34, 35) and electro‐mechanics^36^ equations have been described in detail previously. Both electrophysiolgy and mechanics FE solvers were validated previously in N‐version benchmark studies.(37, 38)

## 3 RESULTS

### 3.1 Verification of FE model

**Figure 5:**
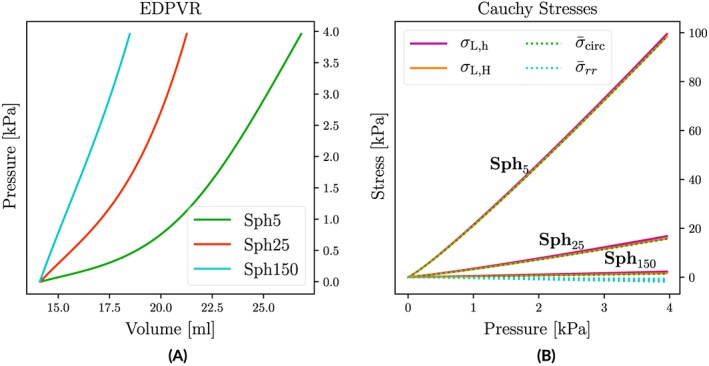
(A) EDPVRs for isotropic spherical shell models of varying wall thickness h. (B) Comparison of FE‐based mean circumferential and radial stresses, ${\bar{\sigma }}_{\;\text{circ}}$ and ${\bar{\sigma }}_{rr}$, with Laplace‐based estimations σ_L,h_ and σ_L,H_

The FE implementation was verified by performing passive inflation experiments with spherical shell models for which Laplace laws are known to be almost exact (**Sph**_5_) or, at least, sufficiently accurate (**Sph**_25_ and **Sph**_150_). The resulting EDPVRs and principal components of the Cauchy stress ***σ***_sph_, evaluated in spherical coordinates and globally averaged to yield mean stresses ${\bar{\sigma }}_{\;\text{circ}}$ and ${\bar{\sigma }}_{rr}$, are shown in Figure 5A,B. A numerical comparison of stresses and work at the maximum pressure of *p* = 4 kPa is provided in Tables 4 and S7.

**Table 4:** Comparison of FE‐based mean wall stresses ${\bar{\sigma }}_{rr}$, ${\bar{\sigma }}_{\theta \theta }$ and ${\bar{\sigma }}_{\varphi \varphi }$ in radial, azimuthal and meridional direction, respectively, with the Laplace‐based wall stress estimates σ_L,h_, σ_L,H_ and σ_L,V_ a

Setup	${\bar{\sigma }}_{rr}$ [kPa]	${\bar{\sigma }}_{\theta \theta }$ [kPa]	${\bar{\sigma }}_{\varphi \varphi }$ [kPa]	***σ***_L,h_ [kPa]	***σ***_L,H_ [kPa]	***σ***_L,V_ [kPa]	#Elements	$\bar{\;dx}$ [mm]
**Sph**_5_	−1.77	99.46	99.47	100.47	99.48		83825	0.65
**Sph**_25_	−1.16	15.63	15.63	16.93	15.99		40974	1.23
**Sph**_150_	−0.63	1.44	1.44	2.35	1.65		213 742	1.70
**LV**_A − Gu_	−0.90	7.05	3.33	5.24	4.40	6.83	420 704	1.52
**LV**_B − Gu_	−0.85	8.71	4.29	5.94	5.08	8.19	332 221	1.74
**LV**_C − Gu_	−0.67	4.47	2.12	3.54	2.76	4.44	456 553	1.84
**LV**_D − Gu_	−0.83	6.97	3.93	5.73	4.88	7.07	394 808	1.86

a All stresses refer to the maximum applied pressure of *p* = 4kPa.

Agreement of FE‐based mean circumferential stress ${\bar{\sigma }}_{\;\text{circ}}$ with Laplace laws was very close, that is, ${\bar{\sigma }}_{\text{circ}}=\frac{1}{2}({\bar{\sigma }}_{\theta \theta }+{\bar{\sigma }}_{\phi \phi })\approx {\bar{\sigma }}_{\theta \theta }\approx {\bar{\sigma }}_{\phi \phi }\approx \sigma_{\text{L,H}}\approx \sigma_{\text{L,h}}$ held. With increasing *h* *σ*_L,H_ provided estimates that were closer to the FE‐based stress ${\bar{\sigma }}_{\;\text{circ}}$ than *σ*_L,h_ (Table 4). The simple Laplace overestimated ${\bar{\sigma }}_{\text{circ}}$ in **Sph**_*5*_, **Sph**_*25*_, and **Sph**_*150*_ by 1.02*%*, 8.32*%*, and 63.19*%*, whereas with *σ*_L,H_, deviations were much smaller with 0.02*%*, 2.3*%*, and 14.58*%*. Radial stresses were negligible in the thinner‐walled models **Sph**_5_ and **Sph**_25_, that is, ${\bar{\sigma }}_{rr}\ll {\bar{\sigma }}_{\text{circ}}$, but not in the thick‐walled model **Sph**_150_ where ${\bar{\sigma }}_{rr}$ amounted to ≈43.75*%* of ${\bar{\sigma }}_{\;\text{circ}}$. A comparison of FE‐based work *W*_int_ to Laplace‐based *W*_int,h_ and *W*_int,H_ is given in Table S7.

### 3.2 Evaluation of Laplace‐based assessment of wall stresses and power

After verification with spherical shell models, FE analysis was applied to a validated in silico EM LV model to compute stresses, power, and work during both diastolic and systolic phases. Since all assumptions underlying Laplace laws are violated in LV models, the FE‐based results were considered the ground truth and, thus, could be used to gauge the accuracy of Laplace‐based assessment of LV mechanics.

#### 3.2.1 Passive inflation of LV models

**Figure 6:**
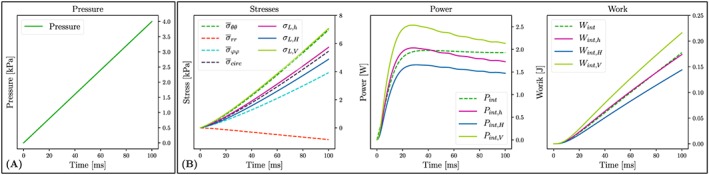
(A) Loading protocol. (B) Stresses, power and work for anisotropic model. Data are shown for model **LV**_D_

Left ventricle models **LV**_*A*_–**LV**_*D*_ were inflated following the same protocol as in section 2.3 (see Figure 6A). The temporal evolution of FE‐ and Laplace‐based stresses, power, and work are shown in Figure 6 for model **LV**_*D*_. Minor quantitative differences to other models **LV**_*A*_–**LV**_*C*_ were observed, but qualitatively, the overall behavior was identical. Stresses at *p* = 4 kPa are summarized in Table 4; incurred work is given in Table S7.

#### 3.2.2 Analysis of LV cycle experiments

**Figure 7:**
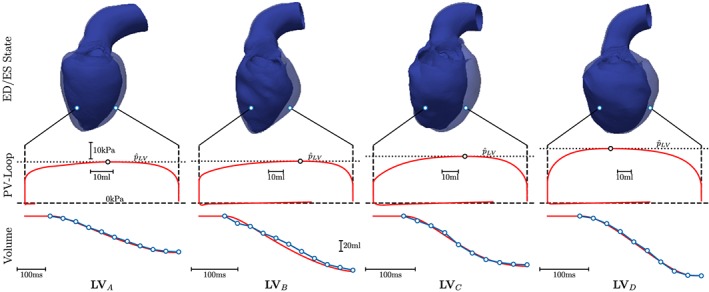
Fitting of EM LV models (red traces) using Cine‐MRI‐based volume data (blue traces) and estimated LV peak pressures, ${\hat{p}}_{\;\text{LV}}$, as input. Top panels show LV anatomy in end‐diastolic (transparent blue) and end‐systolic (solid blue) configuration

Using Cine‐MRI‐based LV volume traces and estimated ${\hat{p}}_{\;\text{LV}}$ as inputs, the models **LV**_*A*_–**LV**_*D*_ were fitted over the cycle phases IVC, ejection, and isovolumetric relaxation (IVR) (Figure 7). All models replicated the clinical metrics of interest such as SV, EF, or peak aortic pressure ${\hat{p}}_{\text{ao}}$ with sufficient accuracy (<5%).

**Figure 8:**
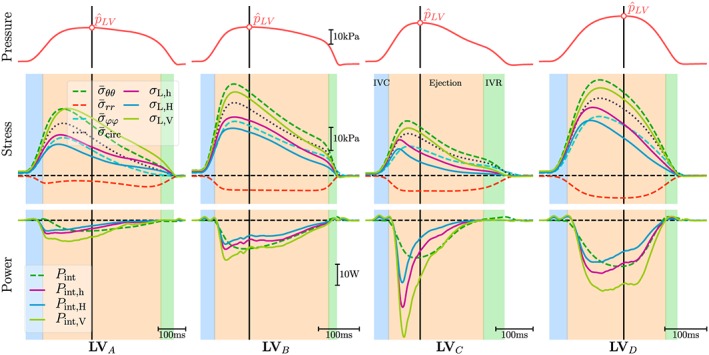
Comparison of FE‐based computation of stresses ( ${\bar{\sigma }}_{\phi \phi }$, ${\bar{\sigma }}_{\theta \theta }$, ${\bar{\sigma }}_{\;\text{circ}}$ and ${\bar{\sigma }}_{rr}$) and power P_int_ with Laplace‐based estimates of stress, σ_L,h_, σ_L,H_ and σ_L,V_, and power P_int,h_, P_int,H_ and P_int,V_. Top panels show the time course of pressure p in the LV endocardium. The solid black vertical line indicates the instant, $t_{\hat{p}}$, when peak pressure in the LV, ${\hat{p}}_{\text{LV}}$, occurs

**Figure 9:**
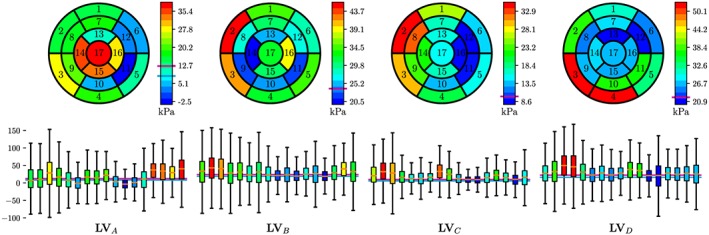
Statistical analysis of the circumferential stress σ_circ_ at the instant of peak pressure. The top row shows σ_circ_ averaged over the corresponding LV segment and the bottom row shows the variation of σ_circ_ in each LV segment

Figure 8 compares the time course of the averaged FE‐based quantities azimuthal, meridionial, radial, and circumferential mean stresses, ${\bar{\sigma }}_{\phi \phi }$, ${\bar{\sigma }}_{\theta \theta }$, ${\bar{\sigma }}_{rr}$, and ${\bar{\sigma }}_{\;\text{circ}}=\frac{1}{2}({\bar{\sigma }}_{\phi \phi }+{\bar{\sigma }}_{\theta \theta })$, respectively, and power *P*_int_ to the Laplace‐based estimation of stresses *σ*_L,⋆_ and power *P*_int,⋆_. In all cases, the Laplace‐based stresses *σ*_L,h_ and *σ*_L,H_ tended to underestimate the FE‐based mean circumferential stress ${\bar{\sigma }}_{\;\text{circ}}$, being closer to the azimuthal stress ${\bar{\sigma }}_{\phi \phi }$, whereas *σ*_L,V_ overestimated ${\bar{\sigma }}_{\text{circ}}$ and was closer to ${\bar{\sigma }}_{\theta \theta }$. Further, both Laplace stresses or globally averaged mean stresses deviate noticeably from the true local stresses acting at a given location (Figure 9).

Laplace‐based power estimates *P*_int,h_, *P*_int,H_, and *P*_int,V_ were qualitatively comparable with the exact FE‐based *P*_int_, but quantitatively marked discrepancies were observed. The time course of Laplace‐based power showed both a faster onset and decay with an early peak in power. Quantitative differences between the Laplace estimates were also significant with *P*_int,V_ > *P*_int,h_ > *P*_int,H_. Around the instant $t_{\hat{p}}$, deviations were in the range of −2.41/ + 2.92, −6.22/ − 0.58, −9.19/ + 9.34, and −7.10/ + 8.28 W for **LV**_*A*_, **LV**_*B*_, **LV**_*C*_, and **LV**_*D*_, respectively. The relative IHP marker led to large deviations (see Figure S13) from the true FE‐based mechanical power, even around the instant of ${\hat{p}}_{\text{LV}}$ the IHP marker was intended for.

A numerical comparison between markers of LV peak power is given in Table 5. Differences between true mechanical peak power ${\hat{P}}_{\text{int}}$ and *P*_int_ at the instant of peak pressure, $t_{\hat{p}}$, were minor, with the maximum difference being $\left|{\hat{P}}_{\text{int}}-P_{\text{int}}(t_{\hat{p}})\right|<0.19$ W or 2.15*%*. Laplace estimation of *P*_int_, evaluated at $t_{\hat{p}}$, misestimated ${\hat{P}}_{\text{int}}$ by 5.1*%* to 54.7*%*. Interestingly, *P*_int,h_ performed better than *P*_int,H_ in all cases, but with nonnegligible maximum relative errors of 23.37*%*, 32.23*%*, 18.51*%*, and 7.68*%* for **LV**_*A*_, **LV**_*B*_, **LV**_*C*_, and **LV**_*D*_, respectively. Internalmyocardial heart power overestimated ${\hat{P}}_{\;\text{int}}$ significantly, in the range between 24.10*%* to 140.28*%*. Since the mechanical power developed during isovolumetric phases was marginal (see Figure S14), the most accurate estimate of *P*_int_ is obtained by computing *P*_ext_ from standard hemodynamic PV data. In terms of peak mechanical power, the difference $\left|{\hat{P}}_{\text{int}}-\hat{P}{}_{\text{ext}}\right|$ was less than 0.5 W or 7.18*%* in all cases where ${\hat{P}}_{\text{ext}}$ can be estimated with high accuracy by taking the product of peak power and flow, ${\hat{p}}_{\text{LV}}\cdot {\hat{q}}_{\text{LV}}$.

**Table 5:** Comparison of FE‐based peak mechanical power ${\hat{P}}_{\text{int}}$ with different estimates that were all evaluated at the instant of peak pressure, $t_{\hat{p}}$: $P_{\text{int}}(t_{\hat{p}})$, $P_{\text{ext}}(t_{\hat{p}})$, $P_{\text{int,h}}(t_{\hat{p}})$, $P_{\text{int,H}}(t_{\hat{p}})$, $P_{\text{int,V}}(t_{\hat{p}})$; peak hydrodynamic power ${\hat{P}}_{\text{ext}}$; product of peak pressure and peak flow in the LV ${\hat{p}}_{\text{LV}}\cdot {\hat{q}}_{\text{LV}}$; relative internal heart power marker IHP and external heart power EHP; cardiac power efficiency P_eff_ and its IHP‐based approximation P_eff,clin_

	${\hat{P}}_{\text{int}}$	$P_{\text{int}}(t_{\hat{p}})$	${\hat{P}}_{\text{ext}}$	$P_{\text{ext}}(t_{\hat{p}})$	${\hat{p}}_{\text{LV}}\cdot {\hat{q}}_{\text{LV}}$	$P_{\text{int,h}}(t_{\hat{p}})$	$P_{\text{int,H}}(t_{\hat{p}})$	$P_{\text{int,V}}(t_{\hat{p}})$	IHP	EHP	*P*_eff_	*P*_eff,clin_
	[W]	[W]	[W]	[W]	[W]	[W]	[W]	[W]	[W]	[W]	[1]	[1]
**LV**_*A*_	−5.13	−5.02	−5.50	−5.42	−5.54	−3.93	−2.62	−7.94	−6.50	−2.56	0.49	0.39
**LV**_*B*_	−13.64	−13.52	−13.56	−13.48	−13.59	−9.24	−7.30	−12.94	−32.76	−6.00	0.43	0.18
**LV**_*C*_	−17.94	−17.75	−17.44	−17.39	−17.48	−14.62	−8.56	−27.09	−22.27	−6.30	0.35	0.28
**LV**_*D*_	−22.01	−21.92	−21.80	−21.74	−21.84	−20.32	−14.82	−30.20	−29.59	−8.02	0.36	0.27

## 4 DISCUSSION

Wall stress and mechanical power generated by the LV are considered important biomarkers that promise potential clinical utility for diagnosis and as a predictor of post‐treatment LV remodeling after interventions.(4, 9) Moreover, the modeling of stresses and power would allow to gain an improved understanding of mechanisms that contribute to adverse remodeling. Laplace analysis would have the charm that inputs such as *p*, *r*, *h*, *V*_myo_, and *V*_cav_ are accessible within routine clinical procedures. However, Laplace analysis is based on a global force balance calculation and relies upon simplifying assumptions on LV shape, tissue structure, and biomechanical behavior. This study attempts to establish validity, accuracy, and potential limitations of Laplace analysis of stresses and mechanical power generated by the LV by comparing against an FE model for which these quantities can be determined with high accuracy.

### 4.1 FE verification

Finite element computation of stresses and mechanical power was verified by performing passive inflation experiments with geometrically well‐defined spherical shell models of varying wall width for which Laplace laws hold with sufficient accuracy. Finite element computed circumferential stresses *σ*_circ_ in all models agreed closely with the Laplace stresses *σ*_L,⋆_ (see Figure 5 and Table 4). As expected, with increasing *h*, deviations became more pronounced, and the thick‐walled Laplace stresses *σ*_L,H_ agreed closer with FE stresses than the standard Laplace stress *σ*_L,h_. In terms of work expended, more noticeable discrepancies were observed between *W*_int_ and Laplace‐based *W*_int,⋆_ (see Table S7). However, since the agreement between FE‐computed internal work *W*_int_ and external work *W*_ext_ was essentially perfect, as expected on grounds of conservation of energy, we concluded that our FE implementation for evaluating stresses, power, and work is correct and that the observed deviations are rather attributable to inherent inaccuracies in the Laplace approximations. In particular, we consider the mean strain rate approximation in Equation 17 and the omission of radial stresses likely candidate causes.

### 4.2 Laplace versus FE‐based stress and power analysis

The validated high‐resolution in silico model served as a reference for evaluating the accuracy of the Laplace‐based approximation of ***σ***, *P*_int_ and *W*_int_. While the FE models that were built from, fitted to, and validated against clinical data may deviate from clinical data within the limits of clinical data uncertainty, for assessing Laplace analysis, the FE model represents the ground truth, as it provides accurate data on stresses ***σ***(**x**,*t*) and strains ***ε***(**x**,*t*), which can serve to compute local power and work densities *p*_int_(**x**,*t*) and *w*_int_(**x**,*t*), respectively, as well as global *P*_int_, *W*_int_, and *W*_ext_ with highest possible accuracy. All input parameters needed for Laplace analysis can be derived from the FE model with higher accuracy than what is achievable clinically. In this regard, the application of Laplace analysis to the in silico model can be considered a best case scenario.

#### 4.2.1 Wall stress in the LV

Wall stress ***σ***(**x**) in the LV is a tensorial quantity that varies in space (see Figure 9). The tensor comprises six independent components, whereas Laplace stresses *σ*_L,⋆_ provide only one scalar stress value representing a global circumferential or hoop stress, *σ*_circ_. While *σ*_circ_ is equivalent to *σ*_*ϕϕ*_ and *σ*_*θθ*_ in a thin‐walled spherical shell such as **Sph**_*5*_ (see Table 4), this is not the case in the LV, as there is no direct equivalence to any component of ***σ***. As shown in an FE modeling study by Zhang et al,^12^ the correlation of Laplace stresses to fiber and cross‐fiber stresses is poor. Conceptually, the force balance consideration used in the derivation of Laplace laws suggests that Laplace stresses are most likely representative of the mean stresses in the longitudinal‐circumferential plane, ${\bar{\sigma }}_{\;\text{circ}}=\frac{1}{2}\left({\bar{\sigma }}_{\phi \phi }+{\bar{\sigma }}_{\theta \theta }\right)$. Indeed, a fair qualitative agreement was observed between ${\bar{\sigma }}_{\text{circ}}$ and *σ*_L,⋆_ during passive LV inflation as illustrated in Figure 6. During ejection, the time course of *σ*_L,⋆_(*t*) followed a similar trend as ${\bar{\sigma }}_{\text{circ}}$ although waveforms deviated to different degrees owing to the marked differences in the LV anatomies. However, quantitatively discrepancies were significant during both passive inflation and over an LV cycle as evident in Figure 6B and in the stress panels of Figure 8 with substantial differences in stress magnitudes between the various Laplace laws and the global circumferential mean stress with $\sigma_{\text{L,V}}>{\bar{\sigma }}_{\text{circ}}>\sigma_{\text{L,h}}>\sigma_{\text{L,H}}$.

Besides the fundamental problem of stress heterogeneity and tensorial properties of LV wall stress, Laplace calculations are afflicted with significant uncertainties. The meaning of geometric parameters *r* and *h* required for the evaluation of Equations 11 or 12 is ambiguous when applied to the LV that deviates in shape markedly from a spherical shell. Therefore, *r* and *h* must be determined from averaging over a number of short axis Cine MRI scans to find representative values. Because of longitudinal shortening, additional averaging occurs, as different slices of the heart are being imaged during ejection. Thus, the determination of parameters *r* and *h* cannot be unique as the particular method employed for averaging, such as the one described in Equation 28, influences, to some extent, the results. Using Equation 13 seems to circumvent this problem since *V*_cav_ and *V*_myo_ are used as inputs that may be determined uniquely for the LV. However, in our simulations, *σ*_L,V_ led to larger misestimations than *σ*_L,h_ and *σ*_L,H_.

It is well known that Laplace‐based calculation of stresses is afflicted with various inaccuracies.^11^ Nonetheless, Laplace‐based calculation of LV wall stresses has been used in clinical studies as a diagnostic criterion.^4^ However, according to observations in this study based on an in silico model and in line with other studies,^12^ the scope for clinical applications appears narrow. Laplace stresses may provide information of diagnostic value, but, if so, rather as an empirical than a mechanistic marker. As a biomarker representing LV wall stresses in a physical sense, Laplace‐based calculations suffer from severe fundamental limitations.

#### 4.2.2 Mechanical heart power and power efficiency

Mechanical heart power *P*_int_ and cardiac power efficiency *P*_eff_ defined as the ratio between peak mechanical power expended by the LV, ${\hat{P}}_{\text{int}}$, and the mean hydrodynamic power delivered to the arterial system, EHP, have been proposed recently as a diagnostic marker.^9^ On grounds of conservation of energy the global mechanical power *P*_int_ expended by the LV and the hydrodynamic power transferred to the LV blood pool, *P*_ext_, must be equal. Discrepancies may occur due to isovolumetric phases during which hydrodynamic power is close to zero, but mechanical power is expended by the LV to some extent as conformational changes of the LV myocardium, and the shape of the LV cavity occur. However, in all models studied, *P*_int_ during isovolumetric phases was negligible (see Figure S14). This does not conflict with experimental studies providing evidence of heterogeneous circumferential strains, longitudinal shortening, and wall thickening during IVC.^39^ Qualitatively similar behavior is observed in our in silico models, but magnitude and velocity of strain development are much smaller during IVC than during ejection. Thus, the strain rate tensors $\dot{\varepsilon }$ remained small during IVC, and mechanical power expenditure was minor. In all LV models under study, global mechanical power *P*_int_ and the hydrodynamic power in the LV cavity *P*_ext_ were virtually identical (see Table 5 and Figure S14). Hence, mechanical heart power can be determined either by analyzing the deformation of the LV myocardium or from PV relations in the LV.

The estimation of *P*_int_ is feasible directly from LV deformation either by using FE models or, as suggested in Fernandes et al,^9^ based on Laplace law where the latter approach is more readily applicable in the clinic. However, when global LV power is of interest, Laplace‐based approaches do not seem to offer any additional benefits over more standard approaches relying on hemodynamic data for a number of reasons.

First of all, the evaluation of *P*_int,⋆_ based on Equation 17 introduces a systematic error that leads to a misestimation of the actual *P*_int_, even in the spherical shell models, since any work expended in the radial direction is ignored. Laplace law takes into account only circumferential stresses and neglects any radial stresses. As shown in Figure 5, this simplification is only well justified in thin‐walled structures such as **Sph**_*5*_ but introduces pronounced discrepancies for increased *h* (see passive inflation experiments in Tables 4 and S6 as well as ${\bar{\sigma }}_{rr}$ traces in Figure 8). Secondly, in addition to the parameters needed for wall stress estimation that are afflicted with substantial uncertainties as discussed above, the parameters *V*_myo_ and $\dot{\varepsilon }$ are required. Using the approximation given by Equation 37 in the Supporting Information, the estimation of ${\dot{\varepsilon }}_{\text{circ}}$ requires that both inner and outer radii *r* and *R* of the LV can be tracked with sufficient temporal resolution and accuracy. However, as evidenced in Figure 8, even when evaluated in an in silico model where tracking of these quantities is feasible with the highest possible accuracy, the overall accuracy of the method is rather poor with significant underestimation or overestimation of the true *P*_int_, depending on whether *P*_int,h_, *P*_int,H_, or *P*_int,V_ is used and whether an early or late phase of ejection is considered (see Figure 8).

The evaluation of cardiac power efficiency *P*_eff_ or *P*_eff,clin_ requires only point estimates of peak mechanical power.

Following Fernandes et al,^9^ this is feasible by assuming that ${\hat{P}}_{\text{int}}$ occurs at the instant of peak pressure, $t_{\hat{p}}$. Consistent with expectations based on Laplace law, this was not the case in any of our LV models. As $p\propto \sigma /\left(r/h\right)$, peak pressure ${\hat{p}}_{\text{LV}}$ and peak stress would only coincide under isometric conditions. In the contracting LV during ejection, the ratio $\left(r/h\right)$ decreases, thus facilitating a further increase of *p* beyond the instant of peak pressure (see pressure and stress panels in Figure 8). Nonetheless, the instants of peak power and peak pressure fell sufficiently close together with $\left|t_{\hat{p}}-t_{\text{pow,peak}}\right|$ of 32, 12, 11, and 9 ms for **LV**_*A*_, **LV**_*B*_, **LV**_*C*_, and **LV**_*D*_, respectively. Indeed, inspection of Table 5 and the power panels in Figure 8 suggest that the Laplace‐based estimation of ${\hat{P}}_{\text{int}}$ seems feasible by evaluating power at the instant of peak pressure (compare ${\hat{P}}_{\;\text{int}}$, $P_{\text{int}}(t_{\hat{p}})$, $P_{\text{int,h}}(t_{\hat{p}}),P_{\text{int,H}}(t_{\hat{p}}),$ and $P_{\text{int,V}}(t_{\hat{p}})$ in Table 5), albeit with inferior accuracy compared with estimations based on hemodynamic PV data.

Alternatively, the simpler *P*_eff,clin_ marker can be used as in Fernandes et al,^9^ which relies on IHP and does not require an estimation of $\dot{\varepsilon }$. While simpler, its use brings about a number of drawbacks. Since $\dot{\varepsilon }$ is ignored, IHP is only a relative marker that is nonlinearly related to *P*_int_. Therefore, IHP provided highly inconsistent relative estimates of *P*_int_ with errors varying in the range from 24.1*%* to 140.28*%* (see Table 5). Thus, IHP as an indicator of *P*_int_ appears to be of insufficient accuracy even for clinical applications of modest accuracy demands. Overall, the scope for Laplace‐based power estimation as proposed in Fernandes et al^9^ seems limited as standard methods based on hemodynamic data are afflicted with less uncertainty, offer higher accuracy, and are easier to evaluate. As shown in Table 5, ${\hat{P}}_{\text{int}}$ is straight forwardly approximated—with higher accuracy than any Laplace‐based method—as the product of peak pressure and flow, $\hat{p}\cdot \hat{q}$.

The mechanical power generated by the LV is an indicator of metabolic demands. Local wall stresses and power densities governing energetic demand and supply ratios in the LV myocardium are known to play important roles as drivers of remodeling in the pressure‐overloaded LV of AS patients. However, analogous to the stresses shown in Figure 8, the distribution of power density *p*_int_(**x**,*t*) in the LV wall is highly heterogeneous as well with significant regional variability around the global mean power density. In this view, Laplace‐based global markers derived from mechanical deformation such as ${\bar{\sigma }}_{\text{circ}}$ or *P*_int,⋆_ are not representative of local stresses and power within the LV myocardium and appear to offer limited insight and predictive power beyond standard PV analysis.

An accurate representation of local mechanical stresses ***σ***(**x**,*t*) and power *p*_int_(**x**,*t*) over a cardiac cycle depends on reliable sets of strains ***ϵ***(**x**,*t*). While techniques for measuring strains in 3D throughout the LV myocardium are available,^14^ such recordings are not part of clinical routine, their analysis requires expensive nontrivial postprocessing, and spatio‐temporal resolution and accuracy are limited. A carefully fitted and validated FE‐based EM LV model that replicates a patients physiology in terms of PV relations as well as LV kinematics provides accurate data on strains ***ϵ***(**x**,*t*) at a high spatio‐temporal resolution. Using an appropriate parameterized patient‐specific constitutive model such as given in Equation 4, ***ϵ***(**x**,*t*) can be used to compute LV wall stresses ***σ***(**x**,*t*) and *P*_int_ or any other stress‐related biomarker efficiently with high accuracy. Such models are able to provide either global power *P*_int_(*t*), but also fine‐grained distributed power density *p*_int_(**x**,*t*). A spatio‐temporal view on ***ε***(**x**,*t*), ***σ***(**x**,*t*), and *p*_int_(**x**,*t*) in the LV may provide additional insights as regions of elevated strain, stress, or power are assumed to be implicated in the mechanisms driving remodeling in the pressure overloaded LV.(40, 41)

## 5 CONCLUSIONS

Laplace estimates of LV wall stress are able to provide a rough approximation of global mean stress in the circumferential‐longitudinal plane of the LV. However, according to FE results, spatial heterogeneity of stresses in the LV wall is significant, leading to major discrepancies between local stresses and global mean stress. Assessment of mechanical power with Laplace methods is feasible, but these are inferior in accuracy compared with FE models and do not offer any benefits compared with standard methods based on hemodynamic data. In this view, the scope for Laplace‐based analysis in clinical applications seems narrow. The accurate assessment of stress and power density distribution in the LV wall is only feasible based on patient‐specific FE modeling.

## Supporting information



Supporting info item
