# Supplementary material for: Assessment of wall stresses and mechanical heart power in the left ventricle: Finite element modeling versus Laplace analysis
Source: Int J Numer Method Biomed Eng. 2018 Sep 30;34(12):e3147. doi: 10.1002/cnm.3147 (PMC6492182; doi:10.1002/cnm.3147)
Supplement: Supplementary file 1 — Supporting info item [file CNM-34-na-s001.pdf]

1 | SUPPLEMENTARY MATERIAL

1.1 | Derivation of Laplace's law

To derive the law of Laplace we consider a spherical shell of inner radius, r and thin walls of a given thickness, h. Inflating the shell by applying a pressure, p, within the shell's cavity, induces deformation which causes the buildup of stresses within the wall. If we consider one half of the sphere, the total force $\mathbf{F}_p$ acting on the inner surface must be balanced with the total force acting over the cut surface (see Fig. 1.A). Due to spherical symmetry, the circumferential stresses $\sigma_{\text{circ}}(\rho)$ at any radius $r \leq \rho \leq R$ must be the same and the shear stress is zero. Integrating circumferential stresses over the cut surface yields the total force balancing the force due to the applied pressure. That is, we have

$$p\, r^2\, \pi \stackrel{!}{=} (R^2 - r^2)\, \sigma_{\text{circ}}\, \pi. \tag{1}$$

Assuming that $h \ll r$, radial stresses are small compared to circumferential stresses, $\sigma_{rr} \ll \sigma_{\text{circ}}$, and the total stress tensor is approximated by

$$\boldsymbol{\sigma} = \begin{pmatrix} \sigma_{xx} & \sigma_{xy} & \sigma_{xz} \\ \sigma_{yx} & \sigma_{yy} & \sigma_{yz} \\ \sigma_{zx} & \sigma_{zy} & \sigma_{zz} \end{pmatrix} = \mathbf{P} \begin{pmatrix} \sigma_{rr} & \sigma_{r\varphi} & \sigma_{r\theta} \\ \sigma_{\varphi r} & \sigma_{\varphi\varphi} & \sigma_{\varphi\theta} \\ \sigma_{\theta r} & \sigma_{\theta\varphi} & \sigma_{\theta\theta} \end{pmatrix} \mathbf{P}^\top \approx \mathbf{P} \begin{pmatrix} 0 & 0 & 0 \\ 0 & \sigma_{\text{circ}} & 0 \\ 0 & 0 & \sigma_{\text{circ}} \end{pmatrix} \mathbf{P}^\top \tag{2}$$

with respect to the spherical coordinate system and the projection matrix $\mathbf{P} = (\mathbf{e}_r, \mathbf{e}_\varphi, \mathbf{e}_\theta)^\top$, see Fig. 1.C. Note that $\boldsymbol{\sigma}$ in a spherical shell differs from a stress tensor in the LV in various ways. Unlike in the LV, stresses in circumferential and meridonial/longitudinal direction are equal whereas in the LV longitudinal stresses tend to be larger than circumferential stresses. Further, the assumption $r \ll h$ is not justified, rather $r \approx h$ holds. Thus radial stresses in the LV are non-negligible, that is, σ_{rr} is at an order of magnitude comparable to σ_{circ}.

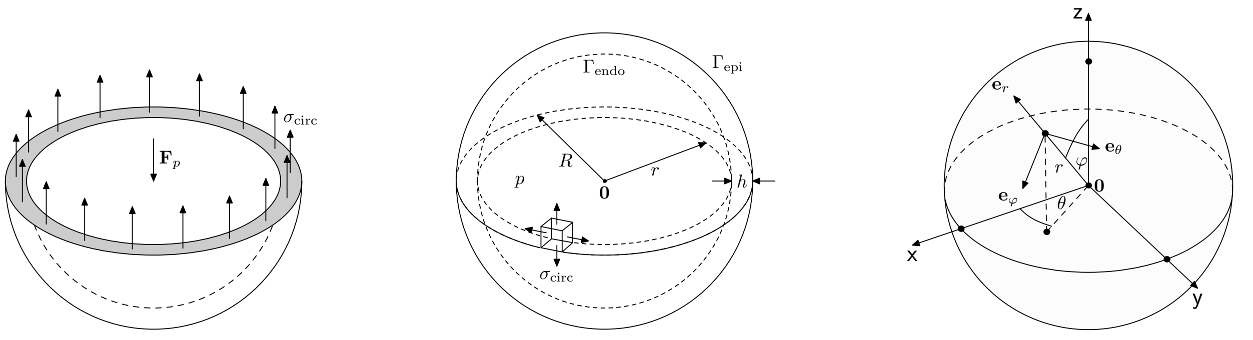

FIGURE 1 Balance of forces in thin-walled spherical shell models, spherical coordinate system and displacement boundary conditions.

1.1.1 | Laplace's law for a thick-walled sphere

From Eq. (1) the circumferential stress in a thick-walled sphere is found as

$$\sigma_{\text{circ}} = \frac{p\, r^2}{(R^2 - r^2)} = \frac{p\, r^2}{(R-r)(R+r)} = \frac{p\, r^2}{h(2\, r + h)} = \frac{p\, r^2}{2\, h\, r\left(1 + \frac{h}{2\, r}\right)} = \frac{p\, r}{2\, h\left(1 + \frac{h}{2\, r}\right)} = \sigma_{\text{L,H}} \tag{3}$$

which we denote as $\sigma_{\text{L,H}}$.

1.1.2 | Laplace's law for a thin-walled sphere

Using assumption **(A3)**, i.e. $h/r \ll 1$, we have $\left(1 + \frac{h}{2\, r}\right) \approx 1$ which yields the simple law of Laplace for a thin-walled sphere given by

$$\sigma_{\text{circ}} = \frac{p\, r}{2\, h\left(1 + \frac{h}{2\, r}\right)} \approx \frac{p\, r}{2\, h} = \sigma_{\text{L,h}} \tag{4}$$

which we denote as $\sigma_{\text{L,h}}$.

1.1.3 | Volume-based stress

Since radius r and wall thickness h are two quantities which are not always available or are hard to determine for a general geometry like a LV, we rewrite Eq. (3) in terms of the cavity volume V_{cav} and the myocardial volume V_{myo}. For the spherical shell geometry there holds $V_{\text{cav}} = \frac{4}{3}\pi r^3$ and $V_{\text{cav}} = \frac{4}{3}\pi\,(R^3 - r^3)$ which entails $r = \left(\frac{3\,V_{\text{cav}}}{4\pi}\right)^{1/3}$ and $(r+h) = \left(\frac{3\,(V_{\text{cav}}+V_{\text{myo}})}{4\pi}\right)^{1/3}$. Using the volume-based representations of r and $r+h$, we can rewrite Eq. (3)

$$\sigma_{\text{L,H}} = \frac{p\,r}{2\,h\left(1+\frac{h}{2\,r}\right)} = \frac{p}{\frac{2\,h}{r}\left(1+\frac{h}{2\,r}\right)} = \frac{p}{\left(\frac{2\,h}{r}+\frac{2\,h^2}{2\,r^2}\right)} = \frac{p}{\left(\frac{r^2}{r^2}+\frac{2\,h\,r}{r^2}+\frac{h^2}{r^2}\right)-1} = \frac{p}{\left(\frac{r+h}{r}\right)^2-1} = \frac{p}{\left(\frac{V_{\text{cav}}+V_{\text{myo}}}{V_{\text{cav}}}\right)^{2/3}-1} = \sigma_{\text{L,V}} \tag{5}$$

which we denote as $\sigma_{\text{L,V}}$.

1.2 | Computation of power and work

The approximations $\sigma_{\text{L,h}}$, $\sigma_{\text{L,H}}$ and $\sigma_{\text{L,V}}$ for the circumferential stress σ_{circ} can be used to derive an estimator for the internal power

$$P_{\text{int}}(t) = \int\limits_{\Omega} \boldsymbol{\sigma}(\mathbf{u},t) : \dot{\boldsymbol{\varepsilon}}(\mathbf{u},t)\;\mathrm{d}\mathbf{x} \tag{6}$$

and internal work

$$W_{\text{int}} = \int\limits_{t_0}^{t} P_{\text{int}}(\tau)d\tau. \tag{7}$$

For this sake, we consider Eq. (6) and the simplified representation of the total stress tensor Eq. 2. In Eq. (6) an approximation of the strain rate $\dot{\boldsymbol{\varepsilon}}$ is required. Rewriting the strain tensor $\boldsymbol{\varepsilon}$ in spherical coordinates, as done for the stress tensor $\boldsymbol{\sigma}$, we obtain

$$\boldsymbol{\varepsilon} = \begin{pmatrix} \varepsilon_{xx} & \varepsilon_{xy} & \varepsilon_{xz} \\ \varepsilon_{yx} & \varepsilon_{yy} & \varepsilon_{yz} \\ \varepsilon_{zx} & \varepsilon_{zy} & \varepsilon_{zz} \end{pmatrix} = \mathbf{P} \begin{pmatrix} \varepsilon_{rr} & \varepsilon_{r\varphi} & \varepsilon_{r\theta} \\ \varepsilon_{\varphi r} & \varepsilon_{\varphi\varphi} & \varepsilon_{\varphi\theta} \\ \varepsilon_{\theta r} & \varepsilon_{\theta\varphi} & \varepsilon_{\theta\theta} \end{pmatrix} \mathbf{P}^{\top}$$

where $\mathbf{P}$ is the projection matrix introduced in Sec. 1.1. Similarly, the strain rate $\dot{\boldsymbol{\varepsilon}}$ is expressed as

$$\dot{\boldsymbol{\varepsilon}} = \mathbf{P} \begin{pmatrix} \dot{\varepsilon}_{rr} & \dot{\varepsilon}_{r\varphi} & \dot{\varepsilon}_{r\theta} \\ \dot{\varepsilon}_{\varphi r} & \dot{\varepsilon}_{\varphi\varphi} & \dot{\varepsilon}_{\varphi\theta} \\ \dot{\varepsilon}_{\theta r} & \dot{\varepsilon}_{\theta\varphi} & \dot{\varepsilon}_{\theta\theta} \end{pmatrix} \mathbf{P}^{\top}.$$

Using Eq. (2), an approximation of the internal power density, p_{int} can be derived as $(\boldsymbol{\sigma} : \dot{\boldsymbol{\varepsilon}}) \approx \sigma_{\text{circ}}\,(\dot{\varepsilon}_{\varphi\varphi} + \dot{\varepsilon}_{\theta\theta})$. Due to the assumption of symmetry **(A2)**, strains in circumferential direction do not vary with space, i,e. $\varepsilon_{\varphi\varphi} = \varepsilon_{\theta\theta} = \varepsilon_{\text{circ}}$, and the approximation for the internal power density simplifies to

$$(\boldsymbol{\sigma} : \dot{\boldsymbol{\varepsilon}}) \approx 2\,\sigma_{\text{circ}}\,\dot{\varepsilon}_{\text{circ}}. \tag{8}$$

An approximation of the circumferential strain $\varepsilon_{\text{circ}}$ can be found based the Cauchy strain and considerations illustrated in Fig. 2. Accordingly, for a given radius r circumferential strain can be approximated as

$$\varepsilon_{\text{circ}} \approx \frac{(l_0+\delta l)-l_0}{l_0} = \frac{(r_0+\delta r)\,\alpha - r_0\,\alpha}{r_0\,\alpha} = \frac{(r_0+\delta r)-r_0}{r_0} = \frac{r}{r_0} - 1$$

and for the circumferential strain rate we obtain $\dot{\varepsilon}_{\text{circ}} \approx \frac{\dot{r}}{r_0}$.

To approximate the circumferential strain rate $\dot{\varepsilon}_{\text{circ}}(\mathbf{u},t)$ of a spherical shell of thickness $h = R - r$, we take the arithmetic mean of the strain rate at inner radius r and outer radius R, that is

$$\dot{\varepsilon}_{\text{circ}}(\mathbf{u},t) \approx \frac{1}{2}\left(\frac{\dot{r}(t)}{r_0} + \frac{\dot{R}(t)}{R_0}\right) \tag{9}$$

where r_0 is the initial inner radius and R_0 is the initial outer radius of the spherical shell at its stress free configuration, i.e. $p = 0$.

Using Eqs. (8) and (9), the internal power can be estimated by

$$\begin{aligned} P_{\text{int}}(t) &= \int\limits_{\Omega} \boldsymbol{\sigma}(\mathbf{u},t) : \dot{\boldsymbol{\varepsilon}}(\mathbf{u},t)\;\mathrm{d}\mathbf{x} \approx V_{\text{myo}}(t)\,2\,\sigma_{\text{circ}}(\mathbf{u},t)\,\frac{1}{2}\left(\frac{\dot{r}(t)}{r_0} + \frac{\dot{R}(t)}{R_0}\right) \\ &= V_{\text{myo}}(t)\,\sigma_{\text{circ}}(\mathbf{u},t)\,\left(\frac{\dot{r}(t)}{r_0} + \frac{\dot{R}(t)}{R_0}\right) \end{aligned}$$

where $V_{\text{myo}}(t)$ is the volume of the shell's wall at time t.

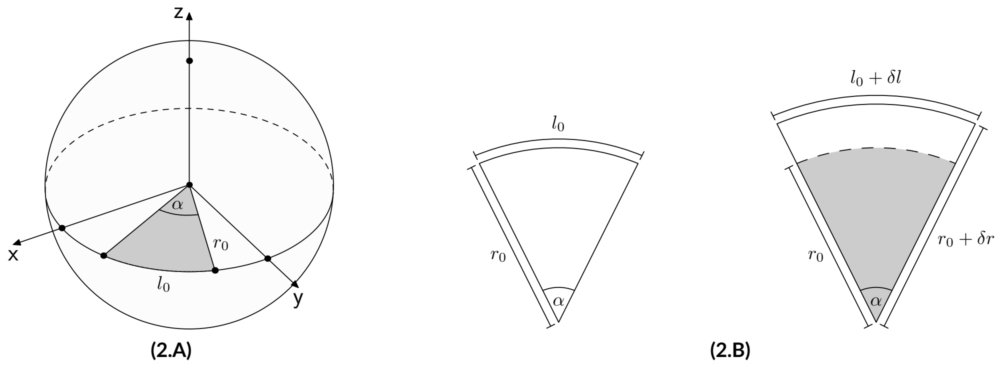

FIGURE 2 Strain estimation.

Using an estimate for σ_{circ} we obtain

$$P_{\text{int},\star}(t) = V_{\text{myo}}(t)\,\sigma_{\text{L},\star}(t)\,\left(\frac{\dot{r}(t)}{r_0} + \frac{\dot{R}(t)}{R_0}\right)$$

with $\star \in \{\text{h}, \text{H}, \text{V}\}$ and by integrating over time, we get an estimate for the internal work

$$W_{\text{int},\star} = \int_0^T P_{\text{int},\star}(t)\;\mathrm{d}t.$$

1.3 | Methods

1.3.1 | Model fitting

To delineate anisotropy from pure geometry effects, passive inflation experiments were also performed with LV models using the Demiray model and passive mechanical behavior was compared to the spherical shell models $\mathbf{Sph}_5$, $\mathbf{Sph}_{25}$and $\mathbf{Sph}_{150}$. In these cases, parameters were set to $b = 7$ and a was chosen in a patient-specific manner to obtain the same volume at maximum inflation pressure as with the Guccione model. Note that in none of the simulations of a full cardiac cycle the Demiray model was considered as the resulting kinematics was in stark contrast to the clinical data.

1.3.2 | Analysis of LV inflation experiments

To evaluate the influence of violating the assumption on the geometry **(A2)**, passive inflation experiments were performed with LV models and the isotropic material due to Demiray,

$$\Psi_{\text{Dem}}(\mathbf{C}) = \frac{\kappa}{2}\,(\log\,J)^2 + \frac{a}{2\,b}\left\{\exp\left[b\left(\operatorname{tr}(\overline{\mathbf{C}}) - 3\right)\right] - 1\right\},$$

following the same protocol as applied to the spherical shell models $\mathbf{Sph}_5$, $\mathbf{Sph}_{25}$ and $\mathbf{Sph}_{150}$. The parameter a in the Demiray model was set to 0.45, 0.63, 0.41 and 0.38 kPa for the models $\mathbf{LV}_{\text{A}}$, $\mathbf{LV}_{\text{B}}$, $\mathbf{LV}_{\text{C}}$ and $\mathbf{LV}_{\text{D}}$, respectively. The Laplace-based stress estimates $\sigma_{\text{L,h}}$, $\sigma_{\text{L,H}}$ and $\sigma_{\text{L,V}}$ were compared to the mean stresses obtained from the FE solution. Stresses were evaluated with respect to an ellipsoidal coordinate system to facilitate a comparison with stresses computed in the spherical shell models $\mathbf{Sph}_5$, $\mathbf{Sph}_{25}$ and $\mathbf{Sph}_{150}$ where spherical coordinates were used for stress analysis. The ellipsoidal coordinate system for the LV models was constructed by assigning fiber and sheet orientations using a rule-based method with a constant fiber angle of $0°$. Stress components $\sigma_{rr}(\mathbf{x})$, $\sigma_{\varphi\varphi}(\mathbf{x})$ and $\sigma_{\theta\theta}(\mathbf{x})$ were averaged yielding $\overline{\sigma}_{rr}$, $\overline{\sigma}_{\varphi\varphi}$ and $\overline{\sigma}_{\theta\theta}$, respectively. Note that all models except $\mathbf{Sph}_5$ showed marked spatial stress variations. Thus, the reported mean stresses $\bar{\sigma}$ may deviate considerably from the true local stresses $\sigma(\mathbf{x})$. Laplace-based estimations of power $P_{\text{int},\star}$ and work $W_{\text{int},\star}$, were compared to those obtained by FE simulation, P_{int} and W_{int} and to external hydrodynamic power and work in the LV cavity, P_{ext} and W_{ext}.

1.4 | Results

1.4.1 | Verification of the FE model

Similarly, with increasing h the accuracy of the thick-walled Laplace estimate $W_{\text{int,H}}$ performed better than the simpler thin-walled Laplace estimate $W_{\text{int,h}}$. As expected on grounds of conservation of energy, the agreement between biomechanical work W_{int} and hemodynamic work W_{ext} was essentially perfect with differences $< 2\%$ for all models.

1.4.2 | Passive inflation of LV models

The LV models $\mathbf{LV}_\mathrm{A}$–$\mathbf{LV}_\mathrm{D}$ were inflated following the loading protocol in Fig. 3.A. Passive material behavior was represented compliant with **(A1)** by the isotropic Demiray model. The temporal evolution of FE- and Laplace-based stresses, power and work are shown in Fig. 3.B for model $\mathbf{LV}_\mathrm{D}$. Minor quantitative differences to other models $\mathbf{LV}_\mathrm{A}$–$\mathbf{LV}_\mathrm{C}$ were observed, but qualitatively the overall behavior was identical. Stresses at $p = 4$ kPa and the amount of work incurred during inflation up to this pressure are summarized in Tabs. 1 and 2.

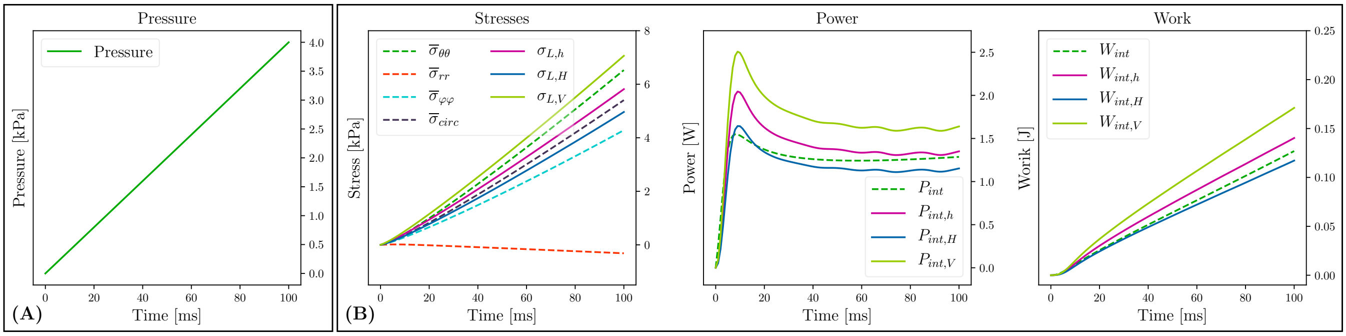

FIGURE 3 (A) Loading protocol. (B) Stresses, power and work for isotropic model. Data are shown for model $\mathbf{LV}_\mathrm{D}$.

Setup	$\overline{\sigma}_{rr}$ [kPa]	$\overline{\sigma}_{\theta\theta}$ [kPa]	$\overline{\sigma}_{\varphi\varphi}$ [kPa]	$\sigma_{\mathrm{L,h}}$ [kPa]	$\sigma_{\mathrm{L,H}}$ [kPa]	$\sigma_{\mathrm{L,V}}$ [kPa]	#elements	$\bar{\mathrm{d}}x$ [mm]
$\mathbf{LV}_{\mathrm{A-Dem}}$	-0.39	6.56	3.77	5.16	4.32	6.81	420704	1.52
$\mathbf{LV}_{\mathrm{B-Dem}}$	-0.38	8.26	4.78	5.92	5.07	8.24	332221	1.74
$\mathbf{LV}_{\mathrm{C-Dem}}$	-0.21	4.07	2.26	3.43	2.65	4.41	456553	1.84
$\mathbf{LV}_{\mathrm{D-Dem}}$	-0.31	6.53	4.28	5.81	4.69	7.06	394808	1.86

TABLE 1 Comparison of FE-based mean wall stresses $\overline{\sigma}_{rr}$, $\overline{\sigma}_{\theta\theta}$ and $\overline{\sigma}_{\varphi\varphi}$ in radial, azimuthal and meridional direction, respectively, with the Laplace-based wall stress estimates $\sigma_{\mathrm{L,h}}$, $\sigma_{\mathrm{L,H}}$ and $\sigma_{\mathrm{L,V}}$. All stresses refer to the maximum applied pressure of $p = 4$ kPa.

Setup	W_{ext} [mJ]	W_{int} [mJ]	$W_{\mathrm{int,h}}$ [mJ]	$W_{\mathrm{int,H}}$ [mJ]	$W_{\mathrm{int,V}}$ [mJ]	#elements	$\bar{\mathrm{d}}x$ [mm]
$\mathbf{Sph}_{5}$	16.82	16.92	19.86	19.65		83825	0.65
$\mathbf{Sph}_{25}$	10.94	10.99	11.58	10.87		40974	1.23
$\mathbf{Sph}_{150}$	8.30	8.15	10.89	7.51		54449	2.31
$\mathbf{LV}_{\mathrm{A-Dem}}$	67.03	65.48	67.94	55.67	89.30	420704	1.52
$\mathbf{LV}_{\mathrm{B-Dem}}$	111.67	110.01	124.41	104.31	183.99	332221	1.74
$\mathbf{LV}_{\mathrm{C-Dem}}$	96.19	94.90	100.48	75.44	128.44	456553	1.84
$\mathbf{LV}_{\mathrm{D-Dem}}$	129.19	126.98	140.30	117.23	171.12	394808	1.86
$\mathbf{LV}_{\mathrm{A-Gu}}$	93.19	89.34	89.11	72.65	116.26	420704	1.52
$\mathbf{LV}_{\mathrm{B-Gu}}$	149.55	145.27	167.23	131.08	219.04	332221	1.74
$\mathbf{LV}_{\mathrm{C-Gu}}$	136.84	133.42	146.17	109.42	183.75	456553	1.84
$\mathbf{LV}_{\mathrm{D-Gu}}$	182.77	177.51	173.92	143.91	216.12	394808	1.86

TABLE 2 Comparison of FE-based biomechanical and hemodynamic work, W_{int} and W_{ext}, with the Laplace-based work estimates $W_{\mathrm{int,h}}$, $W_{\mathrm{int,H}}$ and $W_{\mathrm{int,V}}$ for passive inflation with a pressure of $p = 4$ kPa.

1.4.3 | Analysis of LV cycle experiments

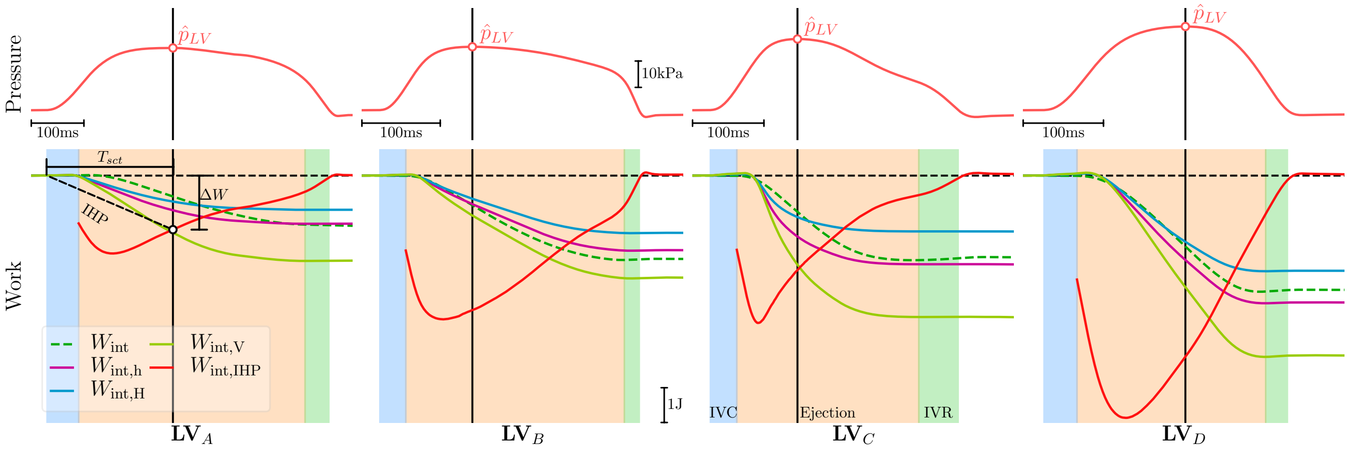

FIGURE 4 Comparison of FE-based computation W_{int} with Laplace-based estimates of work $W_{\text{int,h}}$, $W_{\text{int,H}}$ and $W_{\text{int,V}}$. Top panels show the time course of pressure p in the LV endocardium. The solid black vertical line indicates the instant, $t_{\hat{p}}$, when peak pressure in the LV, $\hat{p}_{\text{LV}}$, occurs.

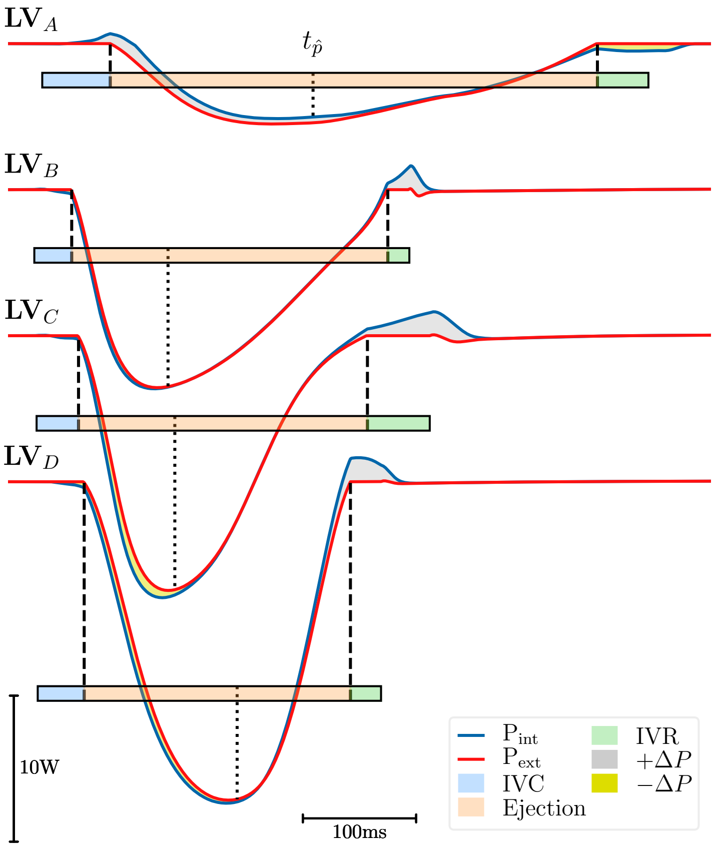

FIGURE 5 Differences between mechanical versus hydrodynamic power, $\Delta P = P_{\text{int}} - P_{\text{ext}}$, during IVC and early ejection were very minor (dark yellow area). A slightly more pronounced ΔP is witnessed during late ejection and IVR (gray area).
